# Antimicrobial usage in broiler poultry farms in Zambia

**DOI:** 10.3389/fvets.2026.1772142

**Published:** 2026-05-12

**Authors:** Isaac Silwamba, John Bwalya Muma, Geoffrey Mainda, NiwaelJesse MtuiMalamsha, Chitwambi Makungu, Kayula Mwila, Tabitha Kimani, Mark Caudell, Mark Obonyo, Charles Bebay, Suze Percy Filippini, Maria Hartmann, Sandra Brogden, Alina Kirse, Lothar Kreienbrock

**Affiliations:** 1 Department of Disease Control, School of Veterinary Medicine, University of Zambia, Lusaka, Zambia; 2 Food and Agriculture Organisation of the United Nations, Lusaka, Zambia; 3 Epidemiology and Information Processing, Institute of Biometry, WHO Collaborating Centre for Research and Training for Health at the Human-Animal-Environment Interface, University of Veterinary Medicine, Hannover, Germany

**Keywords:** antimicrobial resistance, critically important antibiotics, farm level monitoring, treatment frequency, VetCAb-ID

## Abstract

Although Zambia and many other countries globally have very high rates of antimicrobial resistance, there is a surprisingly limited amount of quantitative data on antimicrobial use (AMU) to guide important control actions. Here, we used the VetCAb-ID [Veterinary Consumption of Antibiotics–International Documentation; ©Tierärztliche Hochschule (TiHo) Hannover, Germany] system to conduct a prospective longitudinal analysis of AMU in broiler poultry farms in the Lusaka district of Zambia to quantify the use by the treatment frequency (TF) metric. The overall TF was 7, and the median treatment frequency per day (TFpd) was 0.14. Of all flocks, 80% of them received treatment. Metaphylactic treatment was applied to all flocks at all farms. Overall, a total of nine antimicrobial classes were used, namely tetracyclines, aminoglycosides, aminopenicillins, diaminopyrimidines, fluoroquinolones, macrolides, phosphonic acid derivatives, quinolones, and sulphonamides. Tetracyclines were the antimicrobials most commonly used (62.7%), followed by sulphonamides (15.7%). Prophylactic use accounted for the majority (54.9%) of the total AMU in this study. Therapeutic uses of antimicrobials were primarily for digestive (21.14%) and respiratory (23.43%) problems of the chickens. Of all the treatments recorded, a greater proportion (87.8%) were underdosed, and 7.3% were overdosed. This study demonstrates the feasibility of farm-level monitoring of AMU data from Zambian poultry farms and provides the first quantitative trends of AMU in poultry farms in Zambia. Our findings of the widespread use of antimicrobials for prophylaxis, incorrect dosing patterns, and the relative usage of the highest-priority critically important antimicrobials in Zambian broiler poultry farms are suggestive of misuse. There is a need for continued education about the issue of antimicrobial resistance, application of antimicrobial stewardship, and the establishment of monitoring and benchmarking systems.

## 1 Introduction

Antimicrobial use (AMU) generally refers to the application of substances that destroy or suppress microorganisms. They are widely used to treat infectious diseases in both human and animal medicine as well as agriculture. However, the use, overuse, and misuse of these medicines have led to a serious global public health risk known as antimicrobial resistance (AMR), where microorganisms have evolved to withstand the effects of these medications (1). The majority of antimicrobials used in animals have structural similarities to human medications and have the potential to select for cross-resistance (2). AMR jeopardizes the advancements of modern medicine by increasing mortality, making infections more difficult and expensive to treat, and placing a heavy financial load on healthcare service delivery globally (3).

In contrast to humans, in many countries, AMU in animals is still largely used for mass prophylaxis and growth promotion (4). AMU in animal production is expected to rise by 67% by 2030, primarily because of a shift to intensive farming that regularly uses antimicrobials and an increase in consumer demand for livestock products (5). The use of antimicrobials on farms selects for AMR bacteria, and resistant pathogens can be transferred to humans through contaminated food, direct contact with animals, or indirect environmental mechanisms (6). Additionally, AMU on farms contributes to the presence of antibiotic residues in animal products, which poses several consumer health risks and exacerbates the broader public health issue of antibiotic resistance (7). The community’s low knowledge levels regarding antimicrobial residues, lack of appropriate information regarding the use of antibiotics, and limited residue monitoring systems exacerbate this issue (8).

AMR is higher in environments with the highest levels of AMU because its volume is significantly associated with degrees of AMR (9–11). Although the amount of antimicrobials used by humans and animals is similar in relative terms, new resistant strains are more likely to occur in animals because the biomass of animals raised for food is far higher than that of humans (12). The time scale for resistance to emerge under constant selective pressure is usually much shorter than the decay time following cessation or decline in the volume of drug use. Consequently, significant reductions in resistance necessitate equally significant reductions in AMU (13).

Addressing AMU requires enhanced monitoring, improved biosecurity, and a shift toward more sustainable agricultural practices that promote animal welfare and reduce dependency on antimicrobials (14). AMU monitoring facilitates the monitoring of antimicrobial agent use, identifies trends, and informs public health initiatives (65). Unfortunately, significant data gaps in AMU monitoring still exist despite some good national and regional monitoring systems (15). Around the world, a series of studies dealing with AMU were conducted. Unfortunately, the design and the underlying data structures differ substantially, which restricts the comparability between these studies (16). Only a few studies have been conducted to quantify AMU at the farm level, especially from an African perspective. Furthermore, there are differences in the types of data collected, the methods used for analysis, and the interpretation metrics of the current AMU monitoring data (17).

Metrics for measuring AMU often include quantifying use relative to a population or weights, focusing on dosage, duration, and prescribing trends. However, there is currently no harmonized way to monitor AMU in animals (17). The European Surveillance of Veterinary Antimicrobial Consumption (ESVAC) provides the defined daily dose for animals (DDDvet) and the defined course dose for animals (DCDvet) for antimicrobial agents for use in cattle, pigs, and poultry (18). The DDDvet is the estimated daily average dose per kilogram of animal per species (19, 20). These measures do not exist for Zambia at the moment. Therefore, alternative metrics, such as the used daily dose (UDD) and treatment frequency (TF), have been applied. Both metrics have been used in several countries (21–25). These metrics help to link the use of special animal species. The UDD is the actual average daily dose per kilogram of an animal per species. The ratio of the UDD and the DDDvet can be used to evaluate how the actual AMU compares to a standardized reference dose, and this may ascertain if the antibiotic was overdosed or underdosed (26). In addition, TF can be used for setting up a benchmarking system on a regional level, which was applied in this study (17, 27).

Poultry is a major source of nutrition globally. Given its vast production to meet demand, poultry is one of the livestock species closely linked to excessive AMU (28, 29). AMU in poultry production presents serious problems for both public health safety and animal health management. Although the health of livestock and food animal nutrition depend on antimicrobials, their usage needs to be carefully monitored to reduce the hazards of AMR. In Zambia, broiler poultry farming is a significant contributor to the economy and food security; however, the industry’s reliance on antimicrobials raises concerns about environmental sustainability and public health. It was reported that 86% of layer poultry production used antimicrobials (30). An additional questionnaire study revealed that broiler poultry farmers’ overall average monthly utilization was 48.3% (31). Although neither of these two studies conducted a quantitative analysis of AMU, they did suggest that it might be high in Zambia.

In this work, we applied a longitudinal design to quantify the AMU in Zambian broiler poultry farms using the VetCAb-ID (Veterinary Consumption of Antibiotics–International Documentation; ©TiHo Hannover, Germany) system. We focused on the treatment patterns, classes of antimicrobials used, reasons for AMU, and dosage patterns. Given the high prevalence of AMR in Zambian poultry, our quantitative data on AMU have significance for guiding future research and actions.

## 2 Materials and methods

### 2.1 Study design and ethical approval

We applied a prospective longitudinal design to assess AMU in broiler poultry farms in the Lusaka district of Zambia between 2021 and 2023 across seasonal months. Zambia’s climate is subtropical, with three distinct seasons. We regarded May to July as cold, November to April as wet, and August through October as hot (32). Lusaka was purposely selected as the capital and primary trading hub for poultry products, reflecting common small-to-medium broiler practices in urban/peri-urban Zambia. The snowball sampling approach, in which new units recruit other units to form part of the sample, was used to recruit the participating farms. This approach was used because there was no database register of broiler farms to find the farmers in the study area. Initially, 10 farms were identified through local veterinary and marketplace contacts. Each of the initially selected farms recruited 1 to 4 additional farms. Of the approximately 60 farms that were contacted through this process, 45 enrolled after verbal consent. Farms enrolled in our study had to be active and had to be small to medium-sized (100 to 5,000 birds) farms. We excluded hatcheries, layers/breeders, and village poultry.

Ethical approval was sought from Excellence in Research Ethics and Science (ERES), Ref. No. May-021.

### 2.2 Sampling and data enrollment

A total of 45 small- to medium-sized broiler poultry farms, which largely operated as intensive, were recruited and data on AMU were collected using the VetCAb-ID (©TiHo Hannover, Germany) system after regional adaptation (33). Farms are defined as small- to medium-sized by holding up to 5,000 birds. These farms are usually non- to semi-commercial and focus typically on local markets. At each farm, three flocks were followed longitudinally from day one until the chickens were sold. Weekly follow-ups were carried out using a continuous surveillance-based methodology, and data on the antimicrobial patterns and quantities used, the age group, the dosages, and indications associated with AMU were recorded by the main author. The chicken liveweights necessary to compute antimicrobial dosing metrics were estimated from the chickens’ ages by standardized liveweight tables following Goliomytis et al. (34).

### 2.3 Data analysis

For each of the 135 flocks, we calculated the overall TF and TFpd using Equations 1 and 2 below in order to measure the frequency of antimicrobial treatments during the follow-up period. The TF indicates the average number of days that an animal in the observed population receives treatment throughout a specific period, such as the average number of single doses provided to an individual animal during the observation period (24).

$$\begin{array}{l} \mathrm{TF}\;[\text{treatment frequency}]\\ =\frac{\sum \text{treatments}\;(\#\text{animals treated}\;x\#\text{treatment days})}{\#\text{animals in the population}} \end{array}$$ (1)

$$\begin{array}{l} \text{TFpd}\;[\text{treatment frequency}\;\mathrm{per}\;\mathrm{day}]\\ =\frac{\mathrm{TF}}{\text{duration period}\;(\text{in days})}\; \end{array}$$ (2)

A treatment was considered as applying a single antimicrobial agent to the chickens. Antimicrobial products comprising several antimicrobial ingredients were taken into consideration independently for each antimicrobial class. Coccidiostat-containing treatments were not included in this analysis. Antimicrobial treatments, if any, carried out at the hatchery were also excluded due to a lack of data. Additionally, the UDD for each treatment was calculated. The ESVAC document provided the reference on which DDDvet to use (35). Based on the data from Grave et al., the UDD to DDDvet ratio was also computed, and the appropriateness of dosages was assessed. A correct dosage was defined as a ratio between 0.8 and 1.25, whereas an underdose was defined as a value less than 0.8 and an overdose as a value greater than 1.25 (26). Methods were chosen to enable comparing treatment patterns with other countries.

Separating the TF by means of different antimicrobial classes yield into treatment by class, which was additionally described as relative TF per antimicrobial class, i.e., as a percentage of the TF over the whole fattening period. This period was defined as the period from the general production run until slaughter.

For data management, plausibility control, and data description, respectively, SAS 9.4 M7 (SAS Institute Inc., Cary, NC, United States) was used.

The DDDvet for gentamicin and fosfomycin was challenging to establish. Fosbac, a combination of tylosin and fosfomycin, was one of the antimicrobials used. Tylosin has a DDDvet in the ESVAC list, whereas fosfomycin does not. The tylosin dosage from the packaging insert was out of scope. The choice was to create our own tylosin DDDvet or use the one on the ESVAC list. For gentamicin, the dose information on the back of the packaging insert was provided per ml in drinking water, but we required it in mg/kg live weight. These antibiotics were excluded from the UDD/DDDvet analysis because the ESVAC served as our main source of reference.

## 3 Results

**Figure 1:**
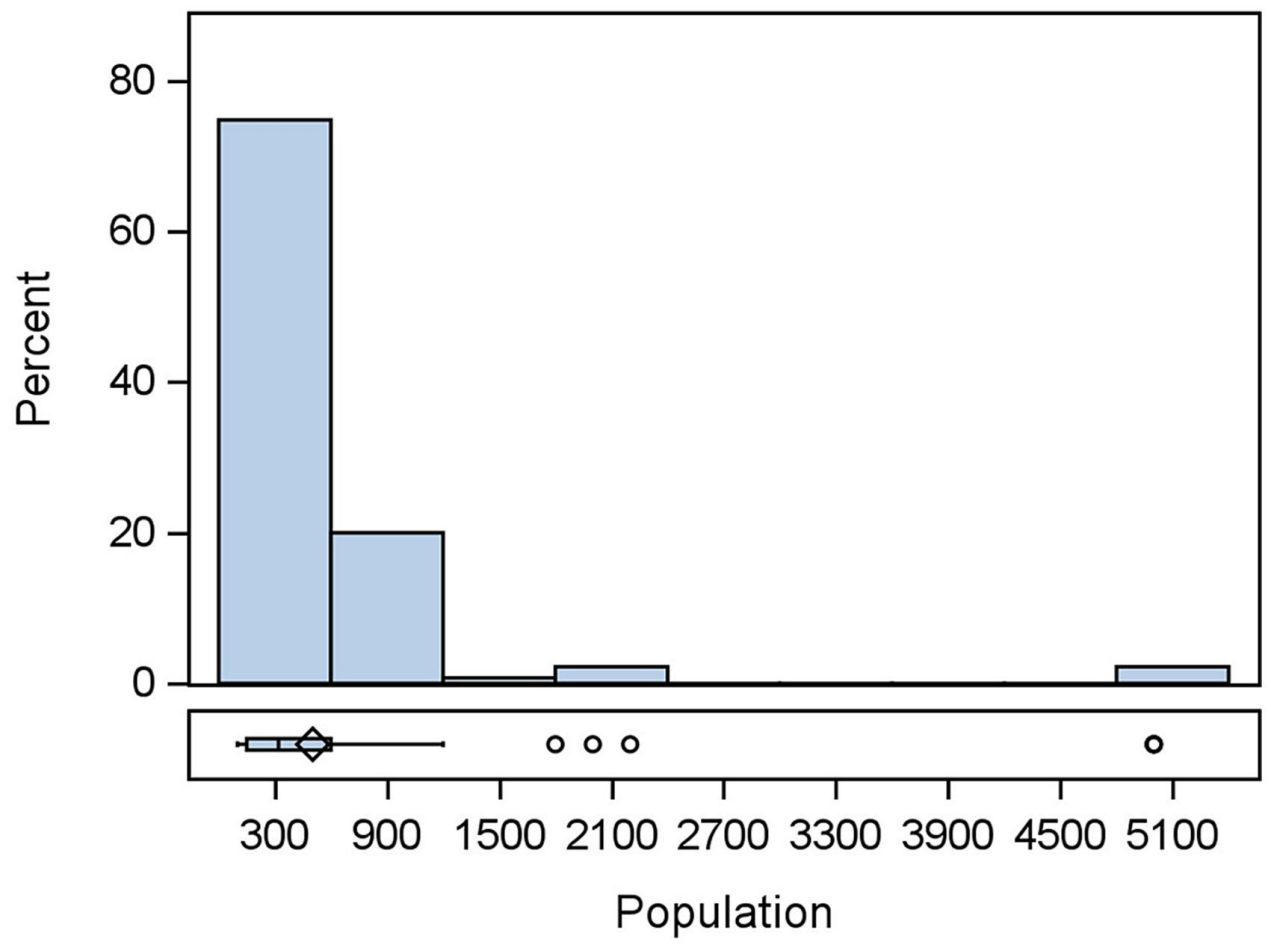
Distribution of population sizes of *n* = 135 flocks.

A total of 135 flocks were studied. The median number of chickens in all flocks was 320, with the minimum being 100 and the maximum being 5,000 (Figure 1).

### 3.1 Treatment patterns

The overall median TF was 7 (lower quartile 3, upper quartile 11.88), with a minimum of 0, a maximum 35, and an inter-quartile-range of 8.88. The median TFpd for each flock was 0.14 (lower quartile 0.07, upper quartile 0.24), with a minimum of 0, a maximum of 0.83, and an inter-quartile-range of 0.17. A total of 80% of the flocks received treatment, while 20% of the flocks did not receive any treatment (Supplementary Table 1).

**Figure 2:**
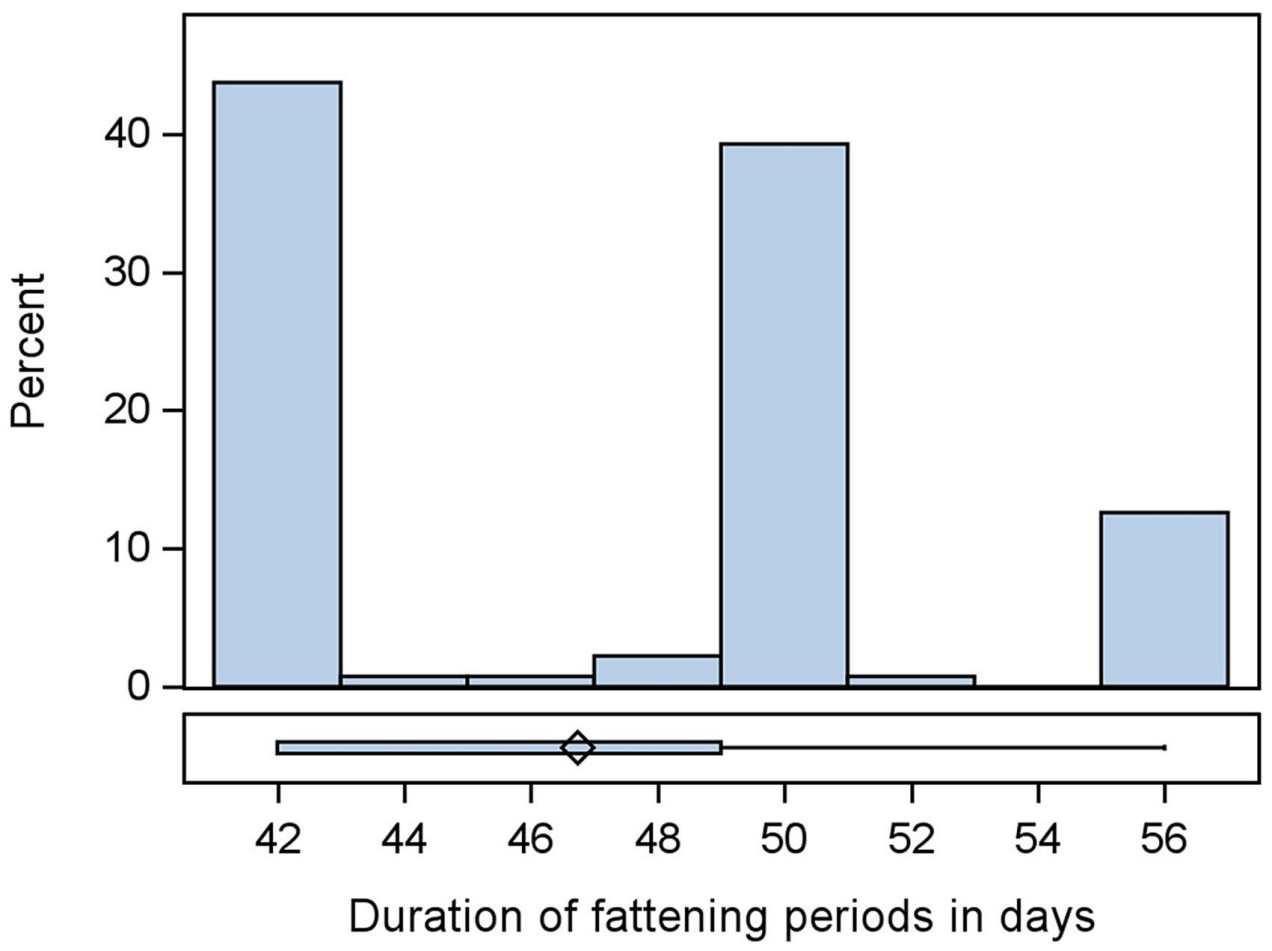
Distribution of duration of fattening periods of *n* = 135 flocks.

**Figure 3:**
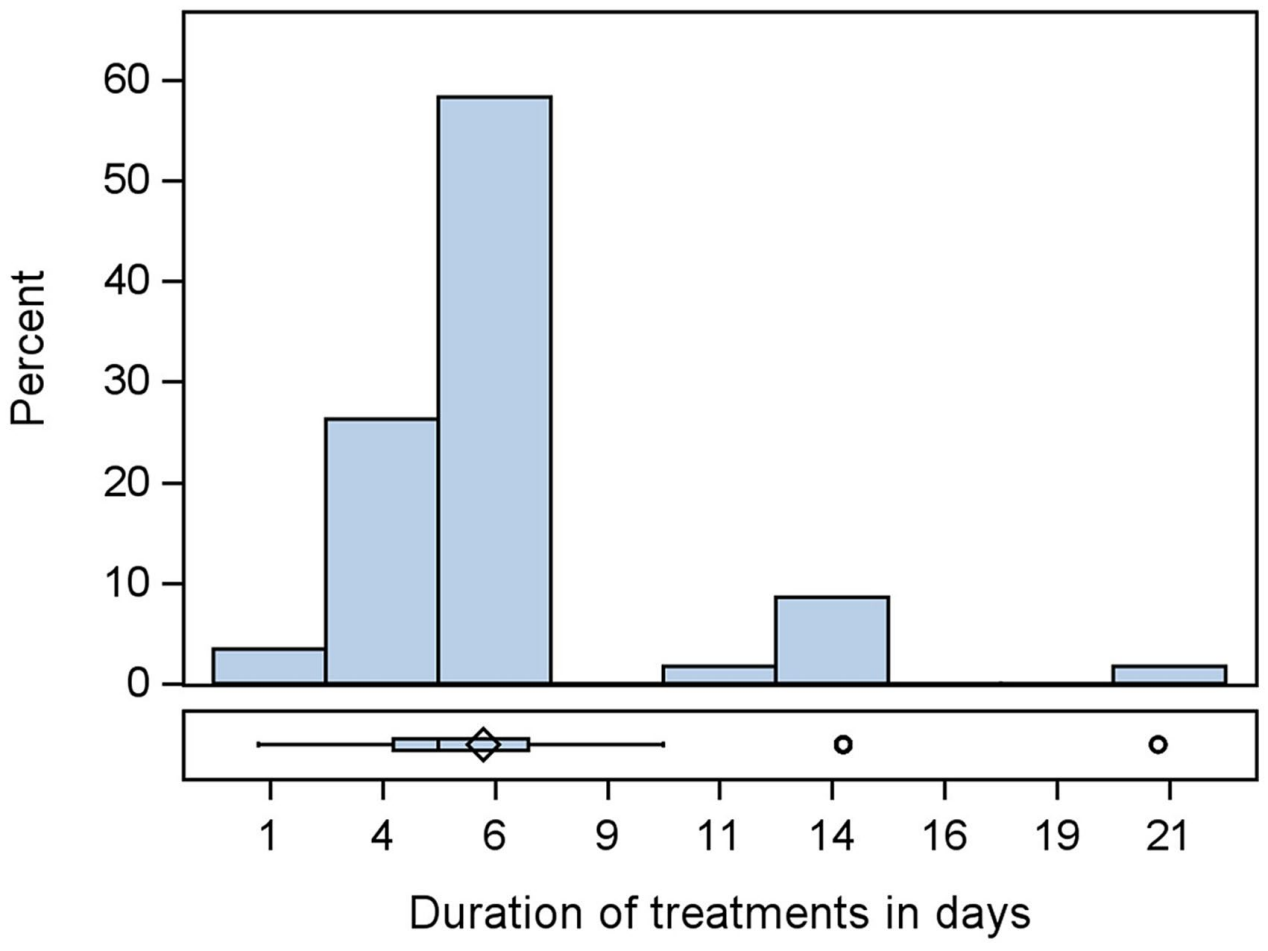
Distribution of duration of treatment of *n* = 135 flocks.

The median duration of the fattening period was 49 days: a minimum of 42 days and a maximum of 56 days (Figure 2). All farmers reported that their treatments were administered orally through drinking water, and the percentage of treated chickens per flock metaphylactically was 100%. The median duration for the treatment of the flocks was 5 days, with a minimum of 1 and a maximum of 21 days (Figure 3). The median of the recorded treatments with consideration of active substances in each flock was 1, with a minimum of 0 and a maximum of 5. The median of the recorded treatment without consideration of the active substance was 0, with a minimum of 0 and a maximum of 3. Of the total flocks, 20% (27/135) of the flocks received no treatment, 54.07% (73/135) received one treatment, 21.48% (29/135) received two treatments, and 4.44% (6/135) received three treatments. Of the 135 flocks, with consideration of the number of active substances in the drugs, 20% (27/135) received no treatment as above; 46.67% (63/135) received one treatment; 21.48% (29/135) received two treatments; 8.15% (11/135) received 3 treatments; 2.96% (4/135) received four treatments; 0.74% (1/135) received five treatments. A higher proportion of treatment was observed in week 1 (49.14%) and week 4 (22.29%) of fattening. Some farmers recorded treatments in week 5 near the cropping weeks of rearing, 8.57% (Supplementary Tables 4–8).

The mean of the distribution of TFpd by the season showed a higher TFpd in the hot season (0.18) compared to the cold (0.17) and wet (0.16) seasons. Farmers who kept flocks of chickens above 1,000 had higher mean TFpd (0.28) compared to farmers who kept below (0.16). Farms with a fattening period above 42 days had higher TFpd (10.70) compared to farms with a fattening period below 42 days (7.32; Supplementary Tables 9–11).

The majority 54.86% of antimicrobial usage was for prophylaxis. Antimicrobial usage for therapeutic purposes was mainly for respiratory 23.43%, digestive 21.14%, and locomotive systems 0.57%. None of the 45 farmers reported antibiotic usage for growth promotion. The antibiotics amoxicillin, fosfomycin, neomycin, oxytetracycline, sulfadiazine, trimethoprim, and tylosin were used for prophylaxis mainly. The antibiotics doxycycline, gentamicin, sulfadimidine, and sulfaquinoxaline were used for digestive tract diseases mainly. The antibiotics enrofloxacin, flumequine, and sulfachlorpyridazine were used for respiratory problems. The chickens were treated for gastrointestinal, locomotive, and respiratory problems at average ages 23, 28, and 22 days, respectively. The chickens were treated for prophylactic reasons for average age 4 days with a minimum age of 1 day and a maximum age of 29 days (Supplementary Tables 12–14).

### 3.2 Classes of antimicrobials used and dosage patterns

A total of nine antimicrobial classes were used, namely, aminoglycosides, aminopenicillins, diaminopyrimidines, fluoroquinolones, macrolides, phosphonic acid derivatives, sulfonamides, and tetracyclines. Of the total antibiotics used, 70.3% contained one active substance while 29.7% contained two active substances (Supplementary Table 15). The most frequently used antimicrobials were tetracyclines (62.7%) and sulfonamides (15.7%). The least used antibiotics were quinolones (0.1%), aminopenicillins (1.3%), and phosphonic acid derivatives (1.0%). The drug name tetracycline (34.3%) was the most frequently used among the tetracycline class, while sulfadimidine (10.9%) was the most frequently used among the sulfonamides (Supplementary Table 16). The TF by antimicrobial class for all 135 flocks is shown in Table 1.

**Table 1:** Distribution of TF by antimicrobial class for n = 135 flocks.

Antimicrobial class	Mean	Median	STD	CV	Min	5% quantile	95% quantile	Max
Aminoglycosides	0.36	0.0	1.5	410.52	0.0	0.0	3.0	10.0
Aminopenicillins	0.10	0.0	0.6	592.25	0.0	0.0	0.0	4.0
Diaminopyrimidines	0.36	0.0	1.5	415.80	0.0	0.0	5.0	10.0
Fluoroquinolones	0.43	0.0	1.4	321.92	0.0	0.0	5.0	7.0
Macrolides	0.32	0.0	1.1	351.12	0.0	0.0	3.0	5.0
Phosphonic acid	0.08	0.0	0.5	581.70	0.0	0.0	0.0	3.0
Quinolones	0.01	0.0	0.1	1161.90	0.0	0.0	0.0	1.0
Sulfonamides	1.22	0.0	2.8	231.25	0.0	0.0	7.0	14.0
Tetracyclines	4.87	5.0	5.1	104.43	0.0	0.0	14.0	21.0

TF, treatment frequency; STD, standard deviation; CV, coefficient of variation; Min, minimum, Max, maximum.

**Figure 4:**
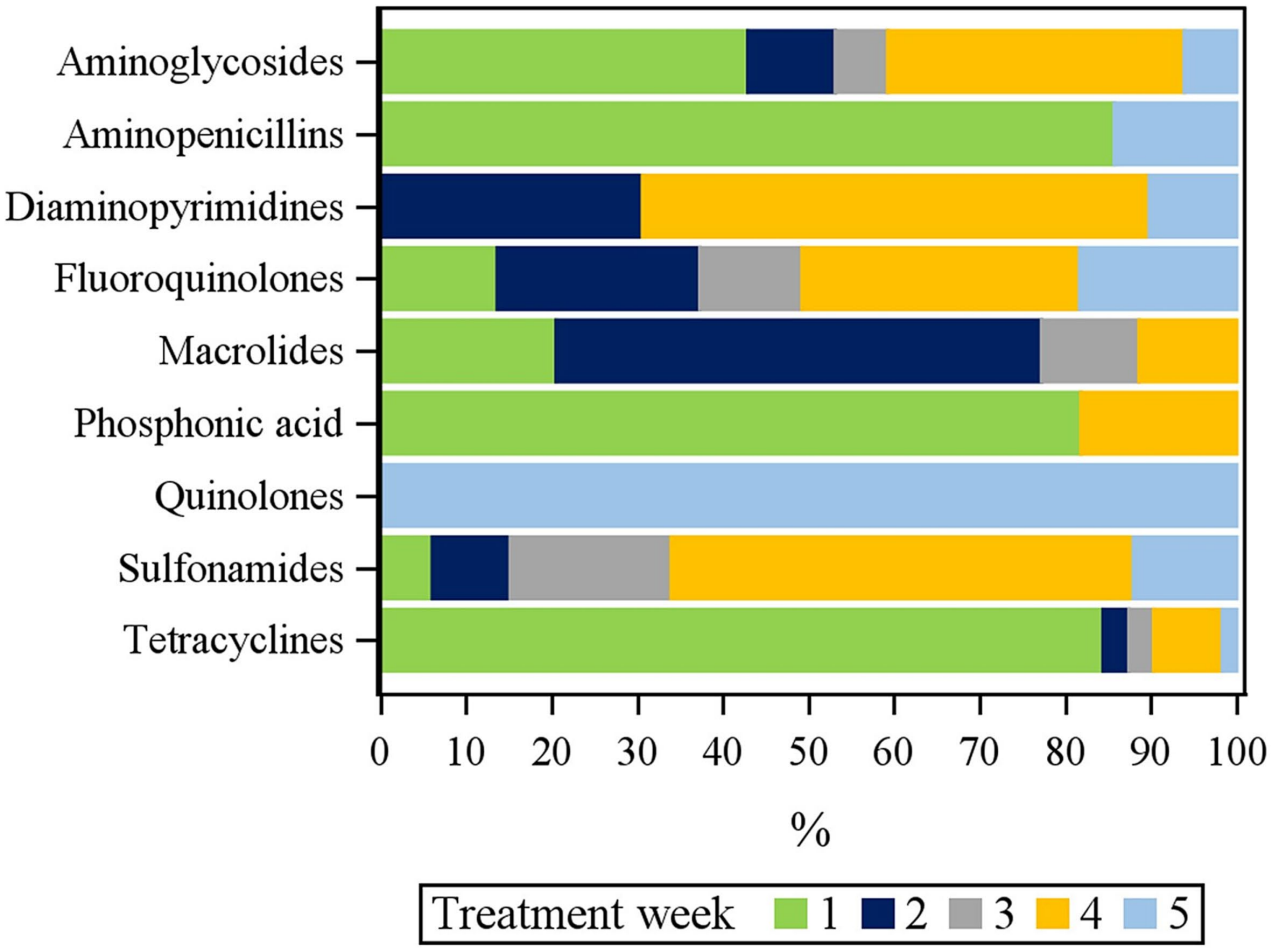
Relative treatment frequency (TF) per antimicrobial class and fattening week for *n* = 135 flocks.

The distribution of antimicrobial classes used by the age of chickens (in days) is also displayed. The aminoglycosides, aminopenicillins, phosphonic acid, and tetracyclines were mostly used in week 1. The macrolide was mostly used in week 2, while diaminopyrimidines were mostly used in week 2 and week 4. The sulfonamides were mostly used in week 4. The quinolones were mostly used in week 5 (Figure 4; Supplementary Table 17).

The UDD and the ratio of the UDD/DDDvet following the ESVAC list were calculated (Table 2) (20). Of all the treatments administered, a proportion of 87.2% were underdosed (Supplementary Tables 19, 20).

**Table 2:** Average UDD from *n* = 135 flocks and DDDvet following ESVAC (20) by antimicrobial component.

ATCvet	ingredient name	DDDvet	UDD	UDD/DDDvet	Total used g	Total used %
QJ01CA04	Amoxicillin	16.0	40.7	2.54	57.2	2.29
QJ01AA02	Doxycycline	15.0	21.5	1.44	188.4	12.57
QJ01MA90	Enrofloxacin	10.0	5.7	0.57	81.5	8.57
QJ01MB07	Flumequin	14.0	0.2	0.01	0.3	0.57
QJ01XX01	Fosfomycin	.	48.2	.	33.0	2.29
QJ01GB03	Gentamicin	.	1.3	.	15.7	4.00
QA07AA51	Neomycin	24.0	1.5	0.06	6.3	1.71
QJ01AA06	Oxytetracycline	39.0	13.6	0.35	163.1	41.14
QJ01EW12	Sulfachlorpyridazine	30.0	17.8	0.59	82.0	2.29
QJ01EW10	Sulfadiazine	34.0	14.9	0.44	63.8	2.29
QJ01EQ03	Sulfadimidine	182.0	24.8	0.14	930.0	10.86
QP51BA02	Sulfaquinoxaline	60.0	2.3	0.04	18.0	0.57
QJ01EW10	Trimethoprim	6.4	3.0	0.46	12.8	2.29
QJ01EW12	Trimethoprim	6.4	3.6	0.56	16.4	2.29
QJ01FA90	Tylosin	81.0	8.5	0.10	68.4	6.29
					1736.7	100.00

UDD, Used Daily Dose; DDDvet, Defined Daily Dose; ESVAC, The European Surveillance of Veterinary Antimicrobial Consumption; ATCvet, Anatomical Therapeutic Chemical classification system for veterinary medicine.

## 4 Discussion

This study aimed to quantify AMU in broiler poultry farms in Zambia and compare the results with those of other countries using the same monitoring system. We quantified the AMU in 45 farms, focusing on the treatment patterns, classes of antimicrobials used, reasons for AMU, and dosage patterns. The overall median TF was 7.0, and the median TFpd was 0.14. This indicates that, on average, the chickens received antimicrobial treatment for 7 days over their total lifespan of 42 days (6 weeks), approximately. This data demonstrates the feasibility of farm-level monitoring AMU from broiler poultry farms in Zambia and remains invaluable in efforts critical for education and raising awareness about the issue of AMR, application of antimicrobial stewardship as well as the establishment of monitoring and benchmarking systems

### 4.1 General treatment patterns

The overall median TF was higher than in Germany (median 6.0) but lower than in poultry farms in Pakistan (median 12.5); (24, 36), all of which were documented in the same system, and therefore compared directly. Out of all flocks, the lowest TF was 0 and the highest was 35. However, some comparisons with the available data of the antibiotic active substances reported in other studies are possible based on the UDD. For instance, in this study, amoxicillin and doxycycline recorded a substantially higher UDD of 40.7 mg/kg and 21.5 mg/kg compared to Pakistan’s 13.2 mg/kg and 5.04 mg/kg, respectively. Enrofloxacin 5.7 mg/kg was higher than a Cameroonian study’s 5 mg/kg, but lower than Pakistan’s 9.51 mg/kg and Tanzania’s 60.8 mg/kg. Compared to studies conducted in Tanzania (135 mg/kg) and Cameroon (36.5 mg/kg), oxytetracycline UDD 13.6 mg/kg in this study was lower, suggesting possible under-dosing or different practices. The sulphonamides, sulfadiazine (14.9 mg/kg) and sulfadimidine (24.8 mg/kg), and Neomycin 1.5 mg/kg were also lower than those reported in the Tanzania (33.8 mg/kg) and Cameroon (21 mg/kg; Neomycin’s 1.5 mg/kg) studies (37–40). Overall, these variations in UDD, particularly for critically essential antibiotics like tetracyclines and fluoroquinolones, suggest variations in stewardship efforts, poultry production scale, or dosage practices in these contexts. We recommend more coordinated research to improve AMU data across different regions.

Of all flocks, 80% received treatment. This is comparable to the AMU of 77–100% observed in other African studies (30–32). All treatments were via drinking water. This is consistent with other studies that reported that the most common method of administering antimicrobials to poultry is the oral route (38, 40–42). However, a Canadian study found that up to 95% of antimicrobials in poultry were given in the feed, which contradicts this finding (43). The frequency of treatments was higher in farms with flocks of more than 1,000 chickens. It has been suggested that the production scale could affect the frequency of AMU (44). A higher proportion of treatments (49.1%) was recorded during the first week of fattening. This is comparable to a study conducted in Europe, which reported that 49% of all AMU was given in the first week, with many farms initiating treatment on the first day of production (45). Higher TF was recorded during the hot season compared to the cold and wet seasons. However, this finding was contradicted by studies conducted in Pakistan, which found that the use of most antimicrobials increased during the cold season as opposed to the hot season (25, 46). Compared to the hot season, the cold season has also been reported to have a higher frequency of respiratory infections (47). TF varied greatly among farms and flocks. Other studies have also reported this finding (45), indicating that further research is needed to fully understand AMU on broiler poultry farms.

Metaphylactic treatment was applied to all flocks at all farms. This practice is relatively common in many poultry settings, particularly due to practical and husbandry challenges associated with separating and treating individual sick chickens (48). If many chickens need to be treated, it takes a lot of time and effort, and it stresses out the personnel and animals (49). However, the primary disadvantage of metaphylactic treatment in poultry, as well as other livestock, is that it may lead to an increase in AMU in general and AMR (28, 50). Some farmers (8.57%) applied treatments closer to the bird maturing dates (weeks five and six) when chickens were supposed to be sold, which could suggest a lack of attention to the withdrawal period. Other studies have also documented this practice of selling chickens while using antibiotics and failing to observe the drug withdrawal periods (40, 41, 51, 52). Only 20% of all flocks received no treatment, which is significantly less than the 31.2% of broiler chicken flocks in the German study that used no antimicrobials at all (24). A study conducted in multiple countries demonstrated that it is possible to raise chickens without antimicrobials, as each country had at least one untreated flock (45). The relatively low percentage of flocks not using antimicrobials in this study may suggest that broiler poultry farms in Zambia heavily rely on the use of antimicrobials. No antibiotic usage for growth promotion was recorded. It has been reported that broilers medicated with antimicrobials generally tend to grow more quickly and consume more feed (53). The lack of AMU for growth promotion in this study may suggest that smallholders in the study group have not popularized this practice.

### 4.2 Treatment by antimicrobial class

A total of nine antimicrobial classes were used, namely, tetracyclines, aminoglycosides, aminopenicillins, diaminopyrimidines, fluoroquinolones, macrolides, phosphonic acid derivatives, quinolones, and sulphonamides. Tetracyclines were the antimicrobials most commonly used (62.7%). This finding was comparable to a number of other studies that found tetracyclines to be the most commonly used antimicrobials in poultry (42, 52, 54, 55). Although tetracycline use appears to be widespread, especially in Sub-Saharan Africa, there is still a lot of diversity in the most often used antimicrobials in poultry. For example, other studies have reported that the most common classes are polymyxins, polypeptides, fluoroquinolones, and sulphonamides (38, 41, 43, 45, 51, 56). These variances in the most commonly used classes may suggest differences in the availability and geographic distribution of these medicines.

Of all the antimicrobials used, 70.3% had only one active ingredient, whereas 29.7% had two. Other studies have also reported the usage of antimicrobials with several active compounds (42, 55, 56). Quinolones were the least used antimicrobials (0.1%). Other studies have also reported this practice of using critically important antimicrobials in poultry (42, 46, 57). The World Health Organisation (WHO) classifies fluoroquinolones, macrolides, and others as “highest priority critically important” antimicrobials (HPCIA), which fits for the low use figure in our study. Of the 175 therapies, 14.9% reported the use of HPICAs. Therefore, the observed relatively low usage of HPICAs (median TF and TFpd < 0.000) in this study may be seen as a good indicator consistent with best practices in antimicrobial stewardship, as overuse of them can increase the development of AMR, undermining their efficacy for the health of humans and animals.

Prophylactic use accounted for the majority (54.86%) of the total AMU in this study. Amoxicillin, fosfomycin, neomycin, oxytetracycline, sulfadiazine, trimethoprim, and tylosin were the most commonly used antibiotics for prophylaxis. The chickens received prophylactic treatment for an average of 4 days, with a minimum age of 1 day and a maximum age of 29 days. Other studies have also demonstrated the widespread usage of these antibiotics, especially tetracyclines or their equivalents, for prophylactic purposes. While other studies have shown the practice of prophylactic antibiotic usage generally, our results are comparatively lower than those of these studies, which found substantially higher rates of prophylactic use, ranging from 78 to 86% (51, 56, 58, 59). However, other studies have also reported the use of antimicrobials mainly for therapeutic rather than prophylactic purposes (38, 40). These variations in the reasons why some farms use antimicrobials primarily for therapeutic purposes while others use them more for prophylactic purposes could suggest variations in access to veterinary services, farming techniques, and animal health management.

Therapeutic uses of antimicrobials were primarily for digestive (21.14%) and respiratory system (23.43%) disorders. Comparable results were reported in another study in South Asia (51). The antimicrobials gentamicin, sulfadimidine, sulfaquinoxaline, and doxycycline were mainly used to treat digestive system disorders, while enrofloxacin, flumequine, and sulfachlorpyridazine were mainly used to treat respiratory system disorders. On average, the chickens received treatment for respiratory, locomotive, and gastrointestinal system disorders in weeks 3 and 4. Tetracyclines, aminoglycosides, phosphonic acid, and aminopenicillins were mostly used in week 1. Diaminopyrimidines were primarily used in weeks 2 and 4, whereas macrolides were primarily used in week 2. The sulphonamides were used most frequently in week 4, while the quinolones were used most frequently in week 5. These variations between different antimicrobial classes and treatment ages were also reported in a German study (24).

Prophylactic antibiotic use was relatively early, frequently during the first week, while treatments for respiratory and gastrointestinal problems were administered mid to near cropping weeks, around 3 to 4 weeks. Understanding these usage patterns can help prevent the overuse of antimicrobials and potentially prevent the development of AMR by ensuring that antimicrobials are only used to treat the infections for which they are intended.

### 4.3 Dosage patterns

As a general result, of all the treatments recorded, a greater proportion 87.8% were underdosed, and 7.3% were overdosed. Other studies have also reported this practice of underdosing and overdosing, mainly exacerbated by a lack of professional support, economic pressures, inadequate regulation, and knowledge gaps in many smallholder poultry production systems. For instance, many farmers lack sufficient understanding about how to use antibiotics properly, and they often do not know the right dosages, frequency, duration, or indications for effective treatment. Smallholder farmers frequently use antibiotics without a prescription or consulting with veterinarians, which results in improper dosage and treatment plans. Farmers may also purposefully administer fewer antibiotics than recommended, dividing doses among more animals or reducing treatment duration in an effort to save costs (40, 60, 61). Due to the possibility that underdosing may promote AMR, we suggest further research to evaluate the levels of AMR and the association between AMU and AMR in this farmer cohort. The finding that certain antibiotics are not on the ESVAC list suggests that some drugs that are not approved for use in Europe are being used in resource-constrained countries.

### 4.4 Pros and cons of the approach

This study primarily compares data from low- and middle-income countries, with some comparisons to industrialized countries due to the limited availability of quantitative AMU data specifically in broiler chickens. The discussion is stratified by geographic regions—Africa, Asia, and high-income countries—to reflect the contextual differences and account for differences in production systems. Therefore, some caution should be applied when evaluating comparisons across studies. Further, data collection in this study was constrained by resources and practical considerations, and certain limitations were identified. The application of a probability-based random sampling was not feasible due to the absence of broiler poultry farm registers and population data. However, this method introduces selection bias into the study as study participants are not selected at random but by referral of already enrolled farms. This may lead to enrolled farms being similar in characteristics. Additionally, some farms did not enroll in the study even though they were contacted due to their reluctance to share AMU records. Farms open to share AMU records may operate differently compared to those that do not want to share. For future studies, a less selection-biased sampling approach would be ideal, however, it might not be possible due to the lack of farm records. Additional caution must be exercised when extrapolating the findings. The inclusion of the farming system in our study was disproportionate. We recommend recruiting participant farms with varying systems using a stratified approach. Lusaka was selected as a typical representative due to its status as a capital city and high poultry activity, which seemed justified. We also assumed that antibiotic use practices would be comparable among broiler small-medium farms throughout the country. Because it is difficult to confirm the accuracy of the farm-level treatment records, quantification data sources have their limitations. Farmers may underreport antibiotic use, deliberately conceal data, or incorrectly report because of fear of regulatory penalties or loss of business (62). In order to address this challenge, farmers were instructed to keep records and antimicrobial product containers for validation. The accuracy and estimation of chicken liveweights, which are necessary to compute antimicrobial dosing metrics, also presented a challenge. To address this challenge, the farmer’s estimation was assumed to be more representative of the actual weights but was cross-checked using theoretical values.

Beyond these limitations, our results are relevant and provide quantitative AMU data collected over a period of more than 2 years using a prospective longitudinal design on a selected number of study farms that are typical of small to-medium-sized broiler production systems in Zambia. Compared to cross-sectional questionnaire studies, longitudinal studies provide more accurate and detailed data because farmers are monitored repeatedly over a specific period (e.g., production cycle); they are also instructed to maintain treatment records and antimicrobial product containers (18). This study demonstrates the feasibility of monitoring AMU data within a globally available database [VetCAb-ID (©TiHo Hannover, Germany)] from Zambian broiler poultry farms and provides the first quantitative trends of AMU in broiler poultry farms in Zambia. Although the status of antimicrobial overuse cannot be determined with certainty, our findings of the widespread use of antimicrobials for prophylaxis, incorrect dosing patterns, and the relative usage of the highest-priority critically important antimicrobials in Zambian broiler poultry farms are suggestive of misuse. There is a need for continued education about the issue of antibiotic resistance, the application of antimicrobial stewardship, and the establishment of monitoring and benchmarking systems. Future research could focus on deciphering the high variation in TF among farms and flocks, as well as the variation in antimicrobial classes used within these settings. It is also crucial to increase research on cost-effective alternative disease prevention strategies and make sure that practical substitutes for AMU are available. Given that high disease incidence remains a significant problem that necessitates AMU (63, 64), farm-level economic sustainability and preservation of food system security must be at the forefront of any future policy initiatives to limit AMU in poultry systems.

## 5 Conclusion

This study demonstrates the feasibility of farm-level monitoring of AMU data from Zambian broiler poultry farms and provides the first quantitative trends of AMU in broiler poultry farms in Zambia. On average, the chickens received antimicrobial treatment for 7 days over their total lifespan. Our findings of the widespread use of antimicrobials for prophylaxis, uncertain dosing patterns, and the relative usage of the highest-priority critically important antimicrobials in Zambian broiler poultry farms are suggestive of misuse. Therefore, there is a continuous need for education about the issue of AMR, application of antimicrobial stewardship, and the establishment of monitoring and benchmarking systems.

## Data Availability

The data were collected on an individual basis from farmers. Each participant provided consent with the understanding that data would not be transferred to any third party. Therefore, data transfer to interested persons is not allowed without an additional formal contract. Data are available to qualified researchers who sign a contract with University of Zambia (UNZA) and TiHo. This contract will include guarantees of the obligation to maintain data confidentiality in accordance with the provisions of the University of Zambia and the European General DataProtection Regulation and its supporting documents in Germany. Currently, there is no data access committee or another body who could be contacted for the data. However, for this purpose, a committee will be founded. This future committee will consist of the authors, as well as other members of UNZA and TiHo. Interested cooperative partners who are able to sign a contract as described above may contact the corresponding author or authors affiliated with the UZNA.
