# Supplementary material for: Antimicrobial usage in broiler poultry farms in Zambia
Source: Front Vet Sci. 2026 May 12;13:1772142. doi: 10.3389/fvets.2026.1772142 (PMC13201229; doi:10.3389/fvets.2026.1772142)
Supplement: Supplementary file 1 [file Table_1.DOCX]

Supplementary Material

# Supplementary Figures and Tables

## Supplementary Tables

Supplementary Table 1: Overall Treatment frequency

|  | N | min | 25%-quantile | median | 75%-quantile | P95 | max | drugs applied during the period | | | |
| --- | --- | --- | --- | --- | --- | --- | --- | --- | --- | --- | --- |
|  |  |  |  |  |  |  |  | yes | | no-nothing | |
|  |  |  |  |  |  |  |  | N | % | N | % |
| TF | 135 | 0.00 | 3.00 | 7.00 | 11.88 | 21.00 | 35.00 | 108 | 80.0 | 27 | 20.0 |
| TFpd | 135 | 0.00 | 0.07 | 0.14 | 0.24 | 0.43 | 0.83 | 108 | 80.0 | 27 | 20.0 |

Abbreviations: TF = treatment frequency; TFpd = treatment frequency per day; N = # of flocks; min = minimum, max = maximum

Supplementary Table 2: Distribution of treatment frequency for each poultry farm

|  | TF | | | | | | | | |
| --- | --- | --- | --- | --- | --- | --- | --- | --- | --- |
|  | N | mean | median | STD | CV | min | 5%-quantile | 95%-quantile | max |
| farm |  |  |  |  |  |  |  |  |  |
| 1 | 3 | 4.33 | 3.00 | 5.13 | 118.42 | 0.00 | 0.00 | 10.00 | 10.00 |
| 2 | 3 | 7.55 | 7.00 | 1.27 | 16.79 | 6.65 | 6.65 | 9.00 | 9.00 |
| 3 | 3 | 8.33 | 10.00 | 2.89 | 34.64 | 5.00 | 5.00 | 10.00 | 10.00 |
| 4 | 3 | 5.00 | 6.00 | 1.73 | 34.64 | 3.00 | 3.00 | 6.00 | 6.00 |
| 5 | 3 | 4.67 | 7.00 | 4.04 | 86.60 | 0.00 | 0.00 | 7.00 | 7.00 |
| 6 | 3 | 7.00 | 7.00 | 0.00 | 0.00 | 7.00 | 7.00 | 7.00 | 7.00 |
| 7 | 3 | 1.67 | 0.00 | 2.89 | 173.21 | 0.00 | 0.00 | 5.00 | 5.00 |
| 8 | 3 | 2.67 | 3.00 | 2.52 | 94.37 | 0.00 | 0.00 | 5.00 | 5.00 |
| 9 | 3 | 35.00 | 35.00 | 0.00 | 0.00 | 35.00 | 35.00 | 35.00 | 35.00 |
| 10 | 3 | 8.00 | 8.00 | 5.00 | 62.50 | 3.00 | 3.00 | 13.00 | 13.00 |
| 11 | 3 | 14.00 | 14.00 | 0.00 | 0.00 | 14.00 | 14.00 | 14.00 | 14.00 |
| 12 | 3 | 17.87 | 18.00 | 0.23 | 1.29 | 17.60 | 17.60 | 18.00 | 18.00 |
| 13 | 3 | 23.00 | 21.00 | 3.46 | 15.06 | 21.00 | 21.00 | 27.00 | 27.00 |
| 14 | 3 | 0.00 | 0.00 | 0.00 | . | 0.00 | 0.00 | 0.00 | 0.00 |
| 15 | 3 | 5.00 | 4.00 | 1.73 | 34.64 | 4.00 | 4.00 | 7.00 | 7.00 |
| 16 | 3 | 6.33 | 7.00 | 1.15 | 18.23 | 5.00 | 5.00 | 7.00 | 7.00 |
| 17 | 3 | 7.67 | 5.00 | 6.43 | 83.86 | 3.00 | 3.00 | 15.00 | 15.00 |
| 18 | 3 | 5.67 | 5.00 | 1.15 | 20.38 | 5.00 | 5.00 | 7.00 | 7.00 |
| 19 | 3 | 6.63 | 4.00 | 4.55 | 68.65 | 4.00 | 4.00 | 11.88 | 11.88 |
| 20 | 3 | 15.00 | 15.00 | 0.00 | 0.00 | 15.00 | 15.00 | 15.00 | 15.00 |
| 21 | 3 | 3.67 | 5.00 | 3.21 | 87.67 | 0.00 | 0.00 | 6.00 | 6.00 |
| 22 | 3 | 0.00 | 0.00 | 0.00 | . | 0.00 | 0.00 | 0.00 | 0.00 |
| 23 | 3 | 13.00 | 8.00 | 8.66 | 66.62 | 8.00 | 8.00 | 23.00 | 23.00 |
| 24 | 3 | 8.00 | 7.00 | 1.73 | 21.65 | 7.00 | 7.00 | 10.00 | 10.00 |
| 25 | 3 | 4.33 | 5.00 | 1.15 | 26.65 | 3.00 | 3.00 | 5.00 | 5.00 |
| 26 | 3 | 3.33 | 0.00 | 5.77 | 173.21 | 0.00 | 0.00 | 10.00 | 10.00 |
| 27 | 3 | 5.33 | 6.00 | 2.08 | 39.03 | 3.00 | 3.00 | 7.00 | 7.00 |
| 28 | 3 | 3.33 | 5.00 | 2.89 | 86.60 | 0.00 | 0.00 | 5.00 | 5.00 |
| 29 | 3 | 6.67 | 10.00 | 5.77 | 86.60 | 0.00 | 0.00 | 10.00 | 10.00 |
| 30 | 3 | 0.00 | 0.00 | 0.00 | . | 0.00 | 0.00 | 0.00 | 0.00 |
| 31 | 3 | 11.93 | 12.00 | 0.12 | 0.97 | 11.80 | 11.80 | 12.00 | 12.00 |
| 32 | 3 | 3.33 | 0.00 | 5.77 | 173.21 | 0.00 | 0.00 | 10.00 | 10.00 |
| 33 | 3 | 4.67 | 7.00 | 4.04 | 86.60 | 0.00 | 0.00 | 7.00 | 7.00 |
| 34 | 3 | 1.67 | 0.00 | 2.89 | 173.21 | 0.00 | 0.00 | 5.00 | 5.00 |
| 35 | 3 | 16.00 | 14.00 | 3.46 | 21.65 | 14.00 | 14.00 | 20.00 | 20.00 |
| 36 | 3 | 14.00 | 14.00 | 0.00 | 0.00 | 14.00 | 14.00 | 14.00 | 14.00 |
| 37 | 3 | 5.00 | 5.00 | 0.00 | 0.00 | 5.00 | 5.00 | 5.00 | 5.00 |
| 38 | 3 | 6.00 | 6.00 | 0.00 | 0.00 | 6.00 | 6.00 | 6.00 | 6.00 |
| 39 | 3 | 2.00 | 3.00 | 1.73 | 86.60 | 0.00 | 0.00 | 3.00 | 3.00 |
| 40 | 3 | 1.33 | 0.00 | 2.31 | 173.21 | 0.00 | 0.00 | 4.00 | 4.00 |
| 41 | 3 | 8.67 | 7.00 | 2.89 | 33.31 | 7.00 | 7.00 | 12.00 | 12.00 |
| 42 | 3 | 14.00 | 14.00 | 0.00 | 0.00 | 14.00 | 14.00 | 14.00 | 14.00 |
| 43 | 3 | 14.00 | 14.00 | 0.00 | 0.00 | 14.00 | 14.00 | 14.00 | 14.00 |
| 44 | 3 | 7.00 | 7.00 | 0.00 | 0.00 | 7.00 | 7.00 | 7.00 | 7.00 |
| 45 | 3 | 7.00 | 7.00 | 0.00 | 0.00 | 7.00 | 7.00 | 7.00 | 7.00 |

Abbreviations: TF = treatment frequency; N = # of flocks; STD = standard deviation; CV = coefficient of variation; min = minimum, max = maximum

Supplementary Table 3: Distribution of TFpd for each poultry farm

|  | TFpd | | | | | | | | |
| --- | --- | --- | --- | --- | --- | --- | --- | --- | --- |
|  | N | mean | median | STD | CV | min | 5%-quantile | 95%-quantile | max |
| farm |  |  |  |  |  |  |  |  |  |
| 1 | 3 | 0.101 | 0.065 | 0.123 | 121.7 | 0.000 | 0.000 | 0.238 | 0.238 |
| 2 | 3 | 0.154 | 0.151 | 0.028 | 18.4 | 0.127 | 0.127 | 0.184 | 0.184 |
| 3 | 3 | 0.198 | 0.238 | 0.069 | 34.6 | 0.119 | 0.119 | 0.238 | 0.238 |
| 4 | 3 | 0.099 | 0.122 | 0.040 | 40.0 | 0.054 | 0.054 | 0.122 | 0.122 |
| 5 | 3 | 0.095 | 0.143 | 0.082 | 86.6 | 0.000 | 0.000 | 0.143 | 0.143 |
| 6 | 3 | 0.156 | 0.167 | 0.019 | 11.9 | 0.135 | 0.135 | 0.167 | 0.167 |
| 7 | 3 | 0.040 | 0.000 | 0.069 | 173.2 | 0.000 | 0.000 | 0.119 | 0.119 |
| 8 | 3 | 0.056 | 0.061 | 0.053 | 95.6 | 0.000 | 0.000 | 0.106 | 0.106 |
| 9 | 3 | 0.833 | 0.833 | 0.000 | 0.0 | 0.833 | 0.833 | 0.833 | 0.833 |
| 10 | 3 | 0.163 | 0.163 | 0.102 | 62.5 | 0.061 | 0.061 | 0.265 | 0.265 |
| 11 | 3 | 0.286 | 0.286 | 0.000 | 0.0 | 0.286 | 0.286 | 0.286 | 0.286 |
| 12 | 3 | 0.370 | 0.367 | 0.004 | 1.1 | 0.367 | 0.367 | 0.374 | 0.374 |
| 13 | 3 | 0.469 | 0.429 | 0.071 | 15.1 | 0.429 | 0.429 | 0.551 | 0.551 |
| 14 | 3 | 0.000 | 0.000 | 0.000 | . | 0.000 | 0.000 | 0.000 | 0.000 |
| 15 | 3 | 0.111 | 0.095 | 0.027 | 24.7 | 0.095 | 0.095 | 0.143 | 0.143 |
| 16 | 3 | 0.113 | 0.125 | 0.021 | 18.2 | 0.089 | 0.089 | 0.125 | 0.125 |
| 17 | 3 | 0.183 | 0.119 | 0.153 | 83.9 | 0.071 | 0.071 | 0.357 | 0.357 |
| 18 | 3 | 0.107 | 0.089 | 0.031 | 28.9 | 0.089 | 0.089 | 0.143 | 0.143 |
| 19 | 3 | 0.122 | 0.083 | 0.078 | 63.8 | 0.071 | 0.071 | 0.212 | 0.212 |
| 20 | 3 | 0.268 | 0.268 | 0.000 | 0.0 | 0.268 | 0.268 | 0.268 | 0.268 |
| 21 | 3 | 0.070 | 0.102 | 0.060 | 86.7 | 0.000 | 0.000 | 0.107 | 0.107 |
| 22 | 3 | 0.000 | 0.000 | 0.000 | . | 0.000 | 0.000 | 0.000 | 0.000 |
| 23 | 3 | 0.265 | 0.163 | 0.177 | 66.6 | 0.163 | 0.163 | 0.469 | 0.469 |
| 24 | 3 | 0.163 | 0.143 | 0.035 | 21.7 | 0.143 | 0.143 | 0.204 | 0.204 |
| 25 | 3 | 0.088 | 0.102 | 0.024 | 26.6 | 0.061 | 0.061 | 0.102 | 0.102 |
| 26 | 3 | 0.068 | 0.000 | 0.118 | 173.2 | 0.000 | 0.000 | 0.204 | 0.204 |
| 27 | 3 | 0.109 | 0.122 | 0.042 | 39.0 | 0.061 | 0.061 | 0.143 | 0.143 |
| 28 | 3 | 0.079 | 0.119 | 0.069 | 86.6 | 0.000 | 0.000 | 0.119 | 0.119 |
| 29 | 3 | 0.119 | 0.179 | 0.103 | 86.6 | 0.000 | 0.000 | 0.179 | 0.179 |
| 30 | 3 | 0.000 | 0.000 | 0.000 | . | 0.000 | 0.000 | 0.000 | 0.000 |
| 31 | 3 | 0.284 | 0.286 | 0.003 | 1.0 | 0.281 | 0.281 | 0.286 | 0.286 |
| 32 | 3 | 0.079 | 0.000 | 0.137 | 173.2 | 0.000 | 0.000 | 0.238 | 0.238 |
| 33 | 3 | 0.095 | 0.143 | 0.082 | 86.6 | 0.000 | 0.000 | 0.143 | 0.143 |
| 34 | 3 | 0.040 | 0.000 | 0.069 | 173.2 | 0.000 | 0.000 | 0.119 | 0.119 |
| 35 | 3 | 0.381 | 0.333 | 0.082 | 21.7 | 0.333 | 0.333 | 0.476 | 0.476 |
| 36 | 3 | 0.333 | 0.333 | 0.000 | 0.0 | 0.333 | 0.333 | 0.333 | 0.333 |
| 37 | 3 | 0.119 | 0.119 | 0.000 | 0.0 | 0.119 | 0.119 | 0.119 | 0.119 |
| 38 | 3 | 0.143 | 0.143 | 0.000 | 0.0 | 0.143 | 0.143 | 0.143 | 0.143 |
| 39 | 3 | 0.048 | 0.071 | 0.041 | 86.6 | 0.000 | 0.000 | 0.071 | 0.071 |
| 40 | 3 | 0.032 | 0.000 | 0.055 | 173.2 | 0.000 | 0.000 | 0.095 | 0.095 |
| 41 | 3 | 0.177 | 0.143 | 0.059 | 33.3 | 0.143 | 0.143 | 0.245 | 0.245 |
| 42 | 3 | 0.333 | 0.333 | 0.000 | 0.0 | 0.333 | 0.333 | 0.333 | 0.333 |
| 43 | 3 | 0.333 | 0.333 | 0.000 | 0.0 | 0.333 | 0.333 | 0.333 | 0.333 |
| 44 | 3 | 0.167 | 0.167 | 0.000 | 0.0 | 0.167 | 0.167 | 0.167 | 0.167 |
| 45 | 3 | 0.143 | 0.143 | 0.000 | 0.0 | 0.143 | 0.143 | 0.143 | 0.143 |

Abbreviations: TFpd = treatment frequency per day ; N = # of flocks; STD = standard deviation; CV = coefficient of variation; min = minimum, max = maximum

Supplementary Table 4: Distribution of chicken count, duration of fattening period and treatment duration

|  | N | mean | median | STD | CV | min | 5%-quantile | 95%-quantile | max | missing |
| --- | --- | --- | --- | --- | --- | --- | --- | --- | --- | --- |
| Number of chickens | 135 | 502.7 | 320.0 | 759.1 | 151.0 | 100.0 | 100.0 | 1200.0 | 5000.0 | 0 |
| Duration of fattening period | 135 | 46.7 | 49.0 | 4.8 | 10.2 | 42.0 | 42.0 | 56.0 | 56.0 | 0 |
| Treatment duraton | 175 | 6.0 | 5.0 | 3.6 | 60.0 | 1.0 | 3.0 | 14.0 | 21.0 | 0 |

Abbreviations: N = # of flocks; STD = standard deviation; CV = coefficient of variation; min = minimum, max = maximum

Supplementary Table 5: Distribution of the number of recorded treatments with and without consideration of active substance

|  | N | mean | median | STD | CV | min | 5%-quantile | 95%-quantile | max |
| --- | --- | --- | --- | --- | --- | --- | --- | --- | --- |
| treatments without consiceration of AS | 149 | 1.1 | 1.0 | 0.8 | 69.4 | 0 | 0.0 | 2.0 | 3 |
| treatments with consiceration of AS | 175 | 1.3 | 1.0 | 1.0 | 78.9 | 0 | 0.0 | 3.0 | 5 |

Abbreviations: AS = active substance; N = # recorded therapies; STD = standard deviation; CV = coefficient of variation; min = minimum, max = maximum

Supplementary Table 6: Total number of recorded treatments

|  | N | % | Total number  of recorded treatments * | %  of recorded treatments |
| --- | --- | --- | --- | --- |
| number of treatments |  |  |  |  |
| 0 | 27 | 20.0 | 0 | 0.0 |
| 1 | 73 | 54.1 | 73 | 49.0 |
| 2 | 29 | 21.5 | 58 | 38.9 |
| 3 | 6 | 4.4 | 18 | 12.1 |
| All | 135 | 100.0 | 149 | 100.0 |

Abbreviations: N = # of flocks. * Total number of recorded treatments = number of treatments x treated flocks

Supplementary Table 7: Total number of recorded treatments with consideration of active substance

|  | N | % | Total number  of recorded treatments* | %  of recorded treatments |
| --- | --- | --- | --- | --- |
| number of treatments |  |  |  |  |
| 0 | 27 | 20.0 | 0 | 0.0 |
| 1 | 63 | 46.7 | 63 | 36.0 |
| 2 | 29 | 21.5 | 58 | 33.1 |
| 3 | 11 | 8.1 | 33 | 18.9 |
| 4 | 4 | 3.0 | 16 | 9.1 |
| 5 | 1 | 0.7 | 5 | 2.9 |
| All | 135 | 100.0 | 175 | 100.0 |

Abbreviations: N = # of flocks. *Total number of recorded treatments = number of treatments x treated flocks

Supplementary Table 8: Distribution of treatment frequency by treatment week

| week | N | % | Cumulative Frequency | Cumulative Percent |
| --- | --- | --- | --- | --- |
| 1 | 86 | 49.14 | 86 | 49.14 |
| 2 | 19 | 10.86 | 105 | 60.00 |
| 3 | 16 | 9.14 | 121 | 69.14 |
| 4 | 39 | 22.29 | 160 | 91.43 |
| 5 | 15 | 8.57 | 175 | 100.00 |

Abbreviations: N = # of treatments

Supplementary Table 9: Distribution of mean daily treatment frequency per flock by the season

| season | Mean TFpd | | | | | | | | | |
| --- | --- | --- | --- | --- | --- | --- | --- | --- | --- | --- |
|  | N | mean | median | STD | CV | min | 5%-quantile | 95%-quantile | max | missing |
| wet | 52 | 0.16 | 0.14 | 0.14 | 84.24 | 0.00 | 0.00 | 0.37 | 0.55 | 0 |
| cold | 40 | 0.17 | 0.14 | 0.15 | 88.65 | 0.00 | 0.00 | 0.38 | 0.83 | 0 |
| hot | 43 | 0.18 | 0.12 | 0.19 | 109.03 | 0.00 | 0.00 | 0.47 | 0.83 | 0 |
| All | 135 | 0.17 | 0.14 | 0.16 | 94.15 | 0.00 | 0.00 | 0.43 | 0.83 | 0 |

Abbreviations: TFpd = treatment frequency per day; N = # of flocks; STD = standard deviation; CV = coefficient of variation; min = minimum, max = maximum

Supplementary Table 10: Distribution of mean daily treatment frequency per flock by the flock size

| flock size | Mean TFpd | | | | | | | | | |
| --- | --- | --- | --- | --- | --- | --- | --- | --- | --- | --- |
|  | N | mean | median | STD | CV | min | 5%-quantile | 95%-quantile | max | missing |
| ≤ 1,000 | 128 | 0.16 | 0.14 | 0.16 | 97.50 | 0.00 | 0.00 | 0.43 | 0.83 | 0 |
| > 1,000 | 7 | 0.28 | 0.33 | 0.13 | 45.21 | 0.13 | 0.13 | 0.48 | 0.48 | 0 |
| All | 135 | 0.17 | 0.14 | 0.16 | 94.15 | 0.00 | 0.00 | 0.43 | 0.83 | 0 |

Abbreviations: TFpd = treatment frequency per day; N = # of flocks; STD = standard deviation; CV = coefficient of variation; min = minimum, max = maximum

Supplementary Table 11: Distribution of mean daily treatment frequency per farm by length fattening period

| fattening period | Mean TFpd | | | | | | | | | |
| --- | --- | --- | --- | --- | --- | --- | --- | --- | --- | --- |
|  | N | mean | median | STD | CV | min | 5%-quantile | 95%-quantile | max | missing |
| ≤ 42 days | 39 | 7.32 | 5.67 | 6.73 | 91.93 | 0.00 | 0.00 | 23.00 | 35.00 | 0 |
| > 42 days | 6 | 10.70 | 10.83 | 5.19 | 48.53 | 3.67 | 3.67 | 17.87 | 17.87 | 0 |
| All | 45 | 7.77 | 6.33 | 6.60 | 84.90 | 0.00 | 0.00 | 17.87 | 35.00 | 0 |

Abbreviations: TFpd = treatment frequency per day; N = # of farms; STD = standard deviation; CV = coefficient of variation; min = minimum, max = maximum

Supplementary Table 12: Distribution of antimicrobial usage by indication for antimicrobial usage

| indication | N | percent |
| --- | --- | --- |
| digestive tract disease* | 37 | 21.14 |
| locomotive system disease | 1 | 0.57 |
| respiratory tract disease* | 41 | 23.43 |
| prophylaxis | 96 | 54.86 |
| All | 175 | 100.00 |

Abbreviations: N = # of treatments. ^*^including glands and appendices

Supplementary Table 13: Distribution of type of active substance and indication for antimicrobial usage

| active substance | indication | | | | | | | | All | |  |
| --- | --- | --- | --- | --- | --- | --- | --- | --- | --- | --- | --- |
|  | digestive tract disease* | | locomotive system disease | | respiratory tract disease* | | prophylaxis | | |  | |
|  | N | % | N | % | N | % | N | % | | N | |
| Amoxicillin | 1 | 25.00 | . | . | . | . | 3 | 75.00 | | 4 | |
| Doxycycline | 8 | 36.36 | 1 | 4.55 | 7 | 31.82 | 6 | 27.27 | | 22 | |
| Enrofloxacin | 3 | 20.00 | . | . | 12 | 80.00 | . | . | | 15 | |
| Flumequin | . | . | . | . | 1 | 100.00 | . | . | | 1 | |
| Fosfomycin | . | . | . | . | 1 | 25.00 | 3 | 75.00 | | 4 | |
| Gentamicin | 5 | 71.43 | . | . | 2 | 28.57 | . | . | | 7 | |
| Neomycin | . | . | . | . | . | . | 3 | 100.00 | | 3 | |
| Oxytetracycline | 6 | 8.33 | . | . | 3 | 4.17 | 63 | 87.50 | | 72 | |
| Sulfachlorpyridazine | 1 | 25.00 | . | . | 3 | 75.00 | . | . | | 4 | |
| Sulfadiazine | . | . | . | . | . | . | 4 | 100.00 | | 4 | |
| Sulfadimidine | 11 | 57.89 | . | . | 4 | 21.05 | 4 | 21.05 | | 19 | |
| Sulfaquinoxaline | 1 | 100.00 | . | . | . | . | . | . | | 1 | |
| Trimethoprim | 1 | 12.50 | . | . | 3 | 37.50 | 4 | 50.00 | | 8 | |
| Tylosin | . | . | . | . | 5 | 45.45 | 6 | 54.55 | | 11 | |
| All | 37 | 21.14 | 1 | 0.57 | 41 | 23.43 | 96 | 54.86 | | 175 | |

Abbreviations: N = # of treatments. ^*^including glands and appendices

Supplementary Table 14: Distribution of age of the treated chickens by treatment indication

| indication | Age in days | | | | | | | | | |
| --- | --- | --- | --- | --- | --- | --- | --- | --- | --- | --- |
|  | N | mean | median | STD | CV | min | 5%-quantile | 95%-quantile | max | missing |
| digestive tract | 37 | 22.57 | 22.00 | 6.98 | 30.94 | 7.00 | 7.00 | 35.00 | 35.00 | 0 |
| locomotive system | 1 | 28.00 | 28.00 | . | . | 28.00 | 28.00 | 28.00 | 28.00 | 0 |
| respiratory tract | 41 | 21.98 | 23.00 | 7.82 | 35.61 | 6.00 | 7.00 | 35.00 | 35.00 | 0 |
| prophylaxis | 96 | 4.01 | 1.00 | 6.61 | 164.71 | 1.00 | 1.00 | 22.00 | 29.00 | 0 |
| All | 175 | 12.28 | 14.00 | 11.48 | 93.48 | 1.00 | 1.00 | 29.00 | 35.00 | 0 |

Abbreviations: N = # of treatments; STD = standard deviation; CV = coefficient of variation; min = minimum, max = maximum

Supplementary Table 15: Distribution of drug names and active substances per drug

|  | | | | | N | % |
| --- | --- | --- | --- | --- | --- | --- |
| substances per drug | antimicrobial class | antimicrobial class id | active substance | active substance id |  |  |
| 1 | Aminopenicillins | 600 | Amoxicillin | 603 | 4 | 2.3 |
|  | Fluoroquinolones | 1800 | Enrofloxacin | 1804 | 15 | 8.6 |
|  | Macrolides | 2300 | Tylosin | 2316 | 3 | 1.7 |
|  | Quinolones | 3500 | Flumequin | 3502 | 1 | 0.6 |
|  | Sulfonamides | 3800 | Sulfadimidine | 3811 | 19 | 10.9 |
|  |  |  | Sulfaquinoxaline | 3825 | 1 | 0.6 |
|  | Tetracyclines | 4000 | Doxycycline | 4002 | 8 | 4.6 |
|  |  |  | Oxytetracycline | 4003 | 12 | 6.9 |
|  |  |  | Tetracycline | 4004 | 60 | 34.3 |
|  | Subtotal | | | | 123 | 70.3 |
| 2 | antimicrobial class | antimicrobial class id | active substance | active substance id |  |  |
|  | Aminoglycosides | 400 | Gentamicin | 406 | 7 | 4.0 |
|  |  |  | Neomycin | 409 | 3 | 1.7 |
|  | Diaminopyrimidines | 1700 | Trimethoprim | 1703 | 8 | 4.6 |
|  | Macrolides | 2300 | Tylosin | 2316 | 8 | 4.6 |
|  | Phosphonic acid derivates | 3000 | Fosfomycin | 3001 | 4 | 2.3 |
|  | Sulfonamides | 3800 | Sulfachlorpyridazine | 3805 | 4 | 2.3 |
|  |  |  | Sulfadiazine | 3807 | 4 | 2.3 |
|  | Tetracyclines | 4000 | Doxycycline | 4002 | 14 | 8.0 |
|  | Subtotal | | | | 52 | 29.7 |
| All | | | | | 175 | 100.0 |

Abbreviations: N = # of treatments

Supplementary Table 16: Distribution of types of antimicrobial classes used

| antimicrobial class | TF-% |
| --- | --- |
| Aminoglycosides | 4.7 |
| Aminopenicillins | 1.3 |
| Diaminopyrimidines | 4.7 |
| Fluoroquinolones | 5.6 |
| Macrolides | 4.2 |
| Phosphonic acid | 1.0 |
| Quinolones | 0.1 |
| Sulfonamides | 15.7 |
| Tetracyclines | 62.7 |
| All | 100.0 |

Abbreviations: TF = treatment frequency

Supplementary Table 17: Distribution of antimicrobial classes used by age of chickens

| antimicrobial class | fattening week | | | | |
| --- | --- | --- | --- | --- | --- |
|  | 1 | 2 | 3 | 4 | 5 |
|  | % | % | % | % | % |
| Aminoglycosides | 42,9 | 10,2 | 6,1 | 34,6 | 6,1 |
| Aminopenicillins | 85,7 | . | . | . | 14,3 |
| Diaminopyrimidines | . | 30,6 | . | 59,2 | 10,2 |
| Fluoroquinolones | 13,6 | 23,7 | 11,9 | 32,4 | 18,3 |
| Macrolides | 20,5 | 56,7 | 11,4 | 11,4 | . |
| Phosphonic_acid | 81,8 | . | . | 18,2 | . |
| Quinolones | . | . | . | . | 100,0 |
| Sulfonamides | 6,1 | 9,1 | 18,8 | 54,0 | 12,1 |
| Tetracyclines | 84,4 | 3,0 | 2,9 | 8,0 | 1,7 |

Supplementary Table 18: Distribution of all treatments, the used daily dose, defined daily dose, and the ratio of the UDD/DDD.

| active substance | treatnent id | amount of drug in g or ml | factor | amount of AS (g or ml) by treatment | number of animals treated | weight of treated animals | treatment days | UDD | DDDvet ESVAC | UDD/DDDvet |
| --- | --- | --- | --- | --- | --- | --- | --- | --- | --- | --- |
| Amoxicillin | 1273 | 100.0 | 0.440 | 44.000 | 900 | 1.3 | 2 | 18.8 | 16 | 1.2 |
| Amoxicillin | 1087 | 10.0 | 0.440 | 4.400 | 400 | 0.06 | 4 | 45.8 | 16 | 2.9 |
| Amoxicillin | 1107 | 10.0 | 0.440 | 4.400 | 400 | 0.06 | 4 | 45.8 | 16 | 2.9 |
| Amoxicillin | 1109 | 10.0 | 0.440 | 4.400 | 350 | 0.06 | 4 | 52.4 | 16 | 3.3 |
| Doxycycline | 1208 | 30.0 | 0.080 | 2.400 | 5000 | 0.04 | 7 | 1.7 | 15 | 0.1 |
| Doxycycline | 1209 | 30.0 | 0.080 | 2.400 | 5000 | 0.04 | 7 | 1.7 | 15 | 0.1 |
| Doxycycline | 1210 | 30.0 | 0.080 | 2.400 | 5000 | 0.04 | 7 | 1.7 | 15 | 0.1 |
| Doxycycline | 1176 | 60.0 | 0.160 | 9.600 | 250 | 0.8 | 5 | 9.6 | 15 | 0.6 |
| Doxycycline | 1178 | 60.0 | 0.160 | 9.600 | 250 | 0.4 | 5 | 19.2 | 15 | 1.3 |
| Doxycycline | 1180 | 60.0 | 0.160 | 9.600 | 240 | 1.4 | 5 | 5.7 | 15 | 0.4 |
| Doxycycline | 1215 | 60.0 | 0.160 | 9.600 | 600 | 0.9 | 3 | 5.9 | 15 | 0.4 |
| Doxycycline | 1216 | 80.0 | 0.160 | 12.800 | 600 | 1.4 | 3 | 5.1 | 15 | 0.3 |
| Doxycycline | 1098 | 100.0 | 0.080 | 8.000 | 800 | 0.9 | 3 | 3.7 | 15 | 0.2 |
| Doxycycline | 1249 | 30.0 | 0.080 | 2.400 | 130 | 1.5 | 3 | 4.1 | 15 | 0.3 |
| Doxycycline | 1250 | 30.0 | 0.080 | 2.400 | 250 | 1.1 | 3 | 2.9 | 15 | 0.2 |
| Doxycycline | 1288 | 100.0 | 0.080 | 8.000 | 2000 | 0.4 | 5 | 2.0 | 15 | 0.1 |
| Doxycycline | 1108 | 10.0 | 0.080 | 0.800 | 394 | 1 | 4 | 0.5 | 15 | 0.0 |
| Doxycycline | 1292 | 100.0 | 0.080 | 8.000 | 400 | 0.9 | 5 | 4.4 | 15 | 0.3 |
| Doxycycline | 1291 | 100.0 | 0.080 | 8.000 | 400 | 0.9 | 5 | 4.4 | 15 | 0.3 |
| Doxycycline | 1115 | 15.0 | 0.400 | 6.000 | 400 | 0.04 | 3 | 125.0 | 15 | 8.3 |
| Doxycycline | 1117 | 15.0 | 0.400 | 6.000 | 400 | 0.04 | 3 | 125.0 | 15 | 8.3 |
| Doxycycline | 1290 | 15.0 | 0.400 | 6.000 | 400 | 0.04 | 3 | 125.0 | 15 | 8.3 |
| Doxycycline | 999 | 5.0 | 0.160 | 0.800 | 146 | 0.6 | 5 | 1.8 | 15 | 0.1 |
| Doxycycline | 1260 | 30.0 | 0.160 | 4.800 | 150 | 0.7 | 5 | 9.1 | 15 | 0.6 |
| Doxycycline | 1263 | 30.0 | 0.160 | 4.800 | 200 | 0.4 | 5 | 12.0 | 15 | 0.8 |
| Doxycycline | 1211 | 400.0 | 0.160 | 64.000 | 5000 | 1.4 | 3 | 3.0 | 15 | 0.2 |
| Enrofloxacin | 1267 | 150.0 | 0.100 | 15.000 | 600 | 1.1 | 5 | 4.5 | 10 | 0.5 |
| Enrofloxacin | 1270 | 120.0 | 0.100 | 12.000 | 600 | 0.7 | 5 | 5.7 | 10 | 0.6 |
| Enrofloxacin | 998 | 15.0 | 0.100 | 1.500 | 146 | 0.6 | 5 | 3.4 | 10 | 0.3 |
| Enrofloxacin | 1259 | 20.0 | 0.100 | 2.000 | 150 | 0.7 | 5 | 3.8 | 10 | 0.4 |
| Enrofloxacin | 1262 | 20.0 | 0.100 | 2.000 | 200 | 0.4 | 5 | 5.0 | 10 | 0.5 |
| Enrofloxacin | 1104 | 5.0 | 0.100 | 0.500 | 150 | 1 | 2 | 1.7 | 10 | 0.2 |
| Enrofloxacin | 1253 | 150.0 | 0.100 | 15.000 | 600 | 1 | 3 | 8.3 | 10 | 0.8 |
| Enrofloxacin | 1254 | 30.0 | 0.100 | 3.000 | 600 | 0.1 | 3 | 16.7 | 10 | 1.7 |
| Enrofloxacin | 1255 | 120.0 | 0.100 | 12.000 | 600 | 0.8 | 3 | 8.3 | 10 | 0.8 |
| Enrofloxacin | 361 | 2.0 | 0.100 | 0.200 | 800 | 1.5 | 3 | 0.1 | 10 | 0.0 |
| Enrofloxacin | 360 | 1.0 | 0.100 | 0.100 | 760 | 2 | 5 | 0.0 | 10 | 0.0 |
| Enrofloxacin | 984 | 2.0 | 0.100 | 0.200 | 100 | 0.18 | 5 | 2.2 | 10 | 0.2 |
| Enrofloxacin | 1265 | 30.0 | 0.100 | 3.000 | 100 | 1 | 3 | 10.0 | 10 | 1.0 |
| Enrofloxacin | 1251 | 120.0 | 0.100 | 12.000 | 600 | 0.7 | 3 | 9.5 | 10 | 1.0 |
| Enrofloxacin | 1252 | 30.0 | 0.100 | 3.000 | 600 | 0.2 | 4 | 6.3 | 10 | 0.6 |
| Flumequin | 1124 | 0.5 | 0.500 | 0.250 | 600 | 2 | 1 | 0.2 | 14 | 0.0 |
| Fosfomycin | 1190 | 15.0 | 0.200 | 3.000 | 250 | 0.08 | 3 | 50.0 | . | . |
| Fosfomycin | 1191 | 15.0 | 0.200 | 3.000 | 250 | 0.08 | 3 | 50.0 | . | . |
| Fosfomycin | 1192 | 15.0 | 0.200 | 3.000 | 250 | 0.08 | 3 | 50.0 | . | . |
| Fosfomycin | 1189 | 120.0 | 0.200 | 24.000 | 200 | 1.4 | 2 | 42.9 | . | . |
| Gentamicin | 1098 | 100.0 | 0.029 | 2.900 | 800 | 0.9 | 3 | 1.3 | . | . |
| Gentamicin | 1249 | 30.0 | 0.029 | 0.870 | 130 | 1.5 | 3 | 1.5 | . | . |
| Gentamicin | 1250 | 30.0 | 0.029 | 0.870 | 250 | 1.1 | 3 | 1.1 | . | . |
| Gentamicin | 1288 | 100.0 | 0.029 | 2.900 | 2000 | 0.4 | 5 | 0.7 | . | . |
| Gentamicin | 1108 | 10.0 | 0.029 | 0.290 | 394 | 1 | 4 | 0.2 | . | . |
| Gentamicin | 1292 | 100.0 | 0.029 | 2.900 | 400 | 0.9 | 5 | 1.6 | . | . |
| Gentamicin | 1291 | 100.0 | 0.050 | 5.000 | 400 | 0.9 | 5 | 2.8 | . | . |
| Neomycin | 1208 | 30.0 | 0.070 | 2.100 | 5000 | 0.04 | 7 | 1.5 | 24 | 0.1 |
| Neomycin | 1209 | 30.0 | 0.070 | 2.100 | 5000 | 0.04 | 7 | 1.5 | 24 | 0.1 |
| Neomycin | 1210 | 30.0 | 0.070 | 2.100 | 5000 | 0.04 | 7 | 1.5 | 24 | 0.1 |
| Oxytetracycline | 1182 | 60.0 | 0.460 | 27.600 | 1000 | 0.9 | 5 | 6.1 | 39 | 0.2 |
| Oxytetracycline | 1184 | 15.0 | 0.460 | 6.900 | 1000 | 0.2 | 5 | 6.9 | 39 | 0.2 |
| Oxytetracycline | 1187 | 10.0 | 0.460 | 4.600 | 320 | 0.04 | 7 | 51.3 | 39 | 1.3 |
| Oxytetracycline | 1084 | 30.0 | 0.910 | 27.300 | 700 | 0.8 | 5 | 9.8 | 39 | 0.3 |
| Oxytetracycline | 1105 | 15.0 | 0.910 | 13.650 | 500 | 0.06 | 3 | 151.7 | 39 | 3.9 |
| Oxytetracycline | 1217 | 40.0 | 0.091 | 3.640 | 500 | 0.9 | 5 | 1.6 | 39 | 0.0 |
| Oxytetracycline | 1103 | 10.0 | 0.182 | 1.820 | 150 | 0.5 | 3 | 8.1 | 39 | 0.2 |
| Oxytetracycline | 1186 | 5.0 | 0.182 | 0.910 | 400 | 0.2 | 5 | 2.3 | 39 | 0.1 |
| Oxytetracycline | 985 | 5.0 | 0.460 | 2.300 | 350 | 0.045 | 21 | 7.0 | 39 | 0.2 |
| Oxytetracycline | 1281 | 5.0 | 0.460 | 2.300 | 350 | 0.04 | 21 | 7.8 | 39 | 0.2 |
| Oxytetracycline | 1282 | 5.0 | 0.460 | 2.300 | 350 | 0.04 | 21 | 7.8 | 39 | 0.2 |
| Oxytetracycline | 1188 | 10.0 | 0.182 | 1.820 | 320 | 0.04 | 7 | 20.3 | 39 | 0.5 |
| Oxytetracycline | 1100 | 15.0 | 0.055 | 0.825 | 530 | 0.045 | 7 | 4.9 | 39 | 0.1 |
| Oxytetracycline | 904 | 5.0 | 0.055 | 0.275 | 170 | 0.04 | 5 | 8.1 | 39 | 0.2 |
| Oxytetracycline | 1245 | 5.0 | 0.055 | 0.275 | 150 | 0.045 | 5 | 8.1 | 39 | 0.2 |
| Oxytetracycline | 1247 | 5.0 | 0.055 | 0.275 | 150 | 0.04 | 5 | 9.2 | 39 | 0.2 |
| Oxytetracycline | 907 | 5.0 | 0.055 | 0.275 | 100 | 0.06 | 7 | 6.5 | 39 | 0.2 |
| Oxytetracycline | 1280 | 10.0 | 0.055 | 0.550 | 100 | 0.05 | 7 | 15.7 | 39 | 0.4 |
| Oxytetracycline | 908 | 45.0 | 0.055 | 2.475 | 2200 | 0.05 | 7 | 3.2 | 39 | 0.1 |
| Oxytetracycline | 1101 | 20.0 | 0.055 | 1.100 | 1200 | 0.055 | 7 | 2.4 | 39 | 0.1 |
| Oxytetracycline | 1277 | 30.0 | 0.055 | 1.650 | 1800 | 0.05 | 7 | 2.6 | 39 | 0.1 |
| Oxytetracycline | 987 | 5.0 | 0.055 | 0.275 | 600 | 0.055 | 3 | 2.8 | 39 | 0.1 |
| Oxytetracycline | 1266 | 5.0 | 0.055 | 0.275 | 600 | 0.05 | 3 | 3.1 | 39 | 0.1 |
| Oxytetracycline | 1268 | 5.0 | 0.055 | 0.275 | 600 | 0.05 | 3 | 3.1 | 39 | 0.1 |
| Oxytetracycline | 1269 | 120.0 | 0.055 | 6.600 | 600 | 0.7 | 5 | 3.1 | 39 | 0.1 |
| Oxytetracycline | 988 | 10.0 | 0.055 | 0.550 | 500 | 0.05 | 14 | 1.6 | 39 | 0.0 |
| Oxytetracycline | 1278 | 10.0 | 0.055 | 0.550 | 350 | 0.05 | 14 | 2.2 | 39 | 0.1 |
| Oxytetracycline | 1279 | 10.0 | 0.055 | 0.550 | 350 | 0.05 | 14 | 2.2 | 39 | 0.1 |
| Oxytetracycline | 997 | 5.0 | 0.055 | 0.275 | 150 | 0.05 | 3 | 12.2 | 39 | 0.3 |
| Oxytetracycline | 1258 | 30.0 | 0.055 | 1.650 | 150 | 0.05 | 3 | 73.3 | 39 | 1.9 |
| Oxytetracycline | 1261 | 30.0 | 0.055 | 1.650 | 200 | 0.05 | 3 | 55.0 | 39 | 1.4 |
| Oxytetracycline | 1000 | 15.0 | 0.055 | 0.825 | 150 | 0.04 | 7 | 19.6 | 39 | 0.5 |
| Oxytetracycline | 1286 | 30.0 | 0.055 | 1.650 | 200 | 0.04 | 7 | 29.5 | 39 | 0.8 |
| Oxytetracycline | 1287 | 15.0 | 0.055 | 0.825 | 150 | 0.04 | 7 | 19.6 | 39 | 0.5 |
| Oxytetracycline | 1001 | 5.0 | 0.055 | 0.275 | 200 | 0.05 | 7 | 3.9 | 39 | 0.1 |
| Oxytetracycline | 1079 | 10.0 | 0.055 | 0.550 | 100 | 0.05 | 7 | 15.7 | 39 | 0.4 |
| Oxytetracycline | 1102 | 10.0 | 0.055 | 0.550 | 120 | 0.05 | 7 | 13.1 | 39 | 0.3 |
| Oxytetracycline | 1085 | 10.0 | 0.055 | 0.550 | 150 | 0.05 | 5 | 14.7 | 39 | 0.4 |
| Oxytetracycline | 1106 | 10.0 | 0.055 | 0.550 | 150 | 0.05 | 5 | 14.7 | 39 | 0.4 |
| Oxytetracycline | 1276 | 10.0 | 0.055 | 0.550 | 100 | 0.05 | 7 | 15.7 | 39 | 0.4 |
| Oxytetracycline | 1119 | 10.0 | 0.055 | 0.550 | 100 | 0.05 | 10 | 11.0 | 39 | 0.3 |
| Oxytetracycline | 1274 | 10.0 | 0.055 | 0.550 | 100 | 0.05 | 7 | 15.7 | 39 | 0.4 |
| Oxytetracycline | 1275 | 10.0 | 0.055 | 0.550 | 100 | 0.04 | 7 | 19.6 | 39 | 0.5 |
| Oxytetracycline | 1120 | 75.0 | 0.055 | 4.125 | 300 | 2 | 5 | 1.4 | 39 | 0.0 |
| Oxytetracycline | 1121 | 60.0 | 0.055 | 3.300 | 300 | 1.5 | 3 | 2.4 | 39 | 0.1 |
| Oxytetracycline | 1257 | 60.0 | 0.055 | 3.300 | 250 | 0.7 | 5 | 3.8 | 39 | 0.1 |
| Oxytetracycline | 1140 | 30.0 | 0.055 | 1.650 | 500 | 0.04 | 5 | 16.5 | 39 | 0.4 |
| Oxytetracycline | 1139 | 30.0 | 0.055 | 1.650 | 500 | 0.04 | 5 | 16.5 | 39 | 0.4 |
| Oxytetracycline | 1175 | 10.0 | 0.055 | 0.550 | 250 | 0.04 | 7 | 7.9 | 39 | 0.2 |
| Oxytetracycline | 1177 | 10.0 | 0.055 | 0.550 | 250 | 0.04 | 7 | 7.9 | 39 | 0.2 |
| Oxytetracycline | 1179 | 10.0 | 0.055 | 0.550 | 250 | 0.04 | 7 | 7.9 | 39 | 0.2 |
| Oxytetracycline | 1212 | 50.0 | 0.055 | 2.750 | 800 | 0.04 | 14 | 6.1 | 39 | 0.2 |
| Oxytetracycline | 1213 | 50.0 | 0.055 | 2.750 | 800 | 0.04 | 14 | 6.1 | 39 | 0.2 |
| Oxytetracycline | 1214 | 50.0 | 0.055 | 2.750 | 800 | 0.04 | 14 | 6.1 | 39 | 0.2 |
| Oxytetracycline | 1193 | 15.0 | 0.055 | 0.825 | 200 | 0.04 | 5 | 20.6 | 39 | 0.5 |
| Oxytetracycline | 1194 | 15.0 | 0.055 | 0.825 | 150 | 0.04 | 5 | 27.5 | 39 | 0.7 |
| Oxytetracycline | 1195 | 15.0 | 0.055 | 0.825 | 150 | 0.04 | 5 | 27.5 | 39 | 0.7 |
| Oxytetracycline | 1196 | 5.0 | 0.055 | 0.275 | 100 | 0.045 | 7 | 8.7 | 39 | 0.2 |
| Oxytetracycline | 1198 | 5.0 | 0.055 | 0.275 | 100 | 0.04 | 7 | 9.8 | 39 | 0.3 |
| Oxytetracycline | 1218 | 5.0 | 0.055 | 0.275 | 100 | 0.04 | 7 | 9.8 | 39 | 0.3 |
| Oxytetracycline | 1199 | 15.0 | 0.055 | 0.825 | 150 | 0.04 | 14 | 9.8 | 39 | 0.3 |
| Oxytetracycline | 1219 | 15.0 | 0.055 | 0.825 | 150 | 0.04 | 14 | 9.8 | 39 | 0.3 |
| Oxytetracycline | 1220 | 15.0 | 0.055 | 0.825 | 150 | 0.04 | 14 | 9.8 | 39 | 0.3 |
| Oxytetracycline | 1200 | 30.0 | 0.055 | 1.650 | 600 | 0.04 | 14 | 4.9 | 39 | 0.1 |
| Oxytetracycline | 1201 | 30.0 | 0.055 | 1.650 | 600 | 0.04 | 14 | 4.9 | 39 | 0.1 |
| Oxytetracycline | 1202 | 30.0 | 0.055 | 1.650 | 600 | 0.04 | 14 | 4.9 | 39 | 0.1 |
| Oxytetracycline | 1203 | 20.0 | 0.055 | 1.100 | 500 | 0.04 | 7 | 7.9 | 39 | 0.2 |
| Oxytetracycline | 1204 | 20.0 | 0.055 | 1.100 | 500 | 0.04 | 7 | 7.9 | 39 | 0.2 |
| Oxytetracycline | 1205 | 20.0 | 0.055 | 1.100 | 500 | 0.04 | 7 | 7.9 | 39 | 0.2 |
| Oxytetracycline | 1206 | 10.0 | 0.055 | 0.550 | 250 | 0.04 | 7 | 7.9 | 39 | 0.2 |
| Oxytetracycline | 1207 | 10.0 | 0.055 | 0.550 | 150 | 0.04 | 7 | 13.1 | 39 | 0.3 |
| Oxytetracycline | 1285 | 10.0 | 0.055 | 0.550 | 150 | 0.04 | 7 | 13.1 | 39 | 0.3 |
| Sulfachlorpyridazine | 1264 | 100.0 | 0.100 | 10.000 | 650 | 0.7 | 5 | 4.4 | 30 | 0.1 |
| Sulfachlorpyridazine | 1293 | 60.0 | 0.400 | 24.000 | 150 | 0.7 | 7 | 32.7 | 30 | 1.1 |
| Sulfachlorpyridazine | 1294 | 60.0 | 0.400 | 24.000 | 200 | 0.9 | 10 | 13.3 | 30 | 0.4 |
| Sulfachlorpyridazine | 1295 | 60.0 | 0.400 | 24.000 | 150 | 1.1 | 7 | 20.8 | 30 | 0.7 |
| Sulfadiazine | 1091 | 45.0 | 0.250 | 11.250 | 300 | 0.4 | 5 | 18.8 | 34 | 0.6 |
| Sulfadiazine | 1111 | 45.0 | 0.250 | 11.250 | 300 | 0.5 | 5 | 15.0 | 34 | 0.4 |
| Sulfadiazine | 1113 | 15.0 | 0.250 | 3.750 | 100 | 0.5 | 5 | 15.0 | 34 | 0.4 |
| Sulfadiazine | 1181 | 150.0 | 0.250 | 37.500 | 500 | 1.4 | 5 | 10.7 | 34 | 0.3 |
| Sulfadimidine | 1099 | 150.0 | 0.930 | 139.500 | 800 | 1.5 | 4 | 29.1 | 182 | 0.2 |
| Sulfadimidine | 358 | 5.0 | 0.930 | 4.650 | 760 | 2 | 2 | 1.5 | 182 | 0.0 |
| Sulfadimidine | 1271 | 200.0 | 0.930 | 186.000 | 900 | 1 | 3 | 68.9 | 182 | 0.4 |
| Sulfadimidine | 1272 | 100.0 | 0.930 | 93.000 | 900 | 1.1 | 4 | 23.5 | 182 | 0.1 |
| Sulfadimidine | 1246 | 30.0 | 0.930 | 27.900 | 150 | 0.9 | 5 | 41.3 | 182 | 0.2 |
| Sulfadimidine | 1248 | 40.0 | 0.930 | 37.200 | 150 | 1.6 | 5 | 31.0 | 182 | 0.2 |
| Sulfadimidine | 905 | 10.0 | 0.930 | 9.300 | 350 | 1 | 3 | 8.9 | 182 | 0.0 |
| Sulfadimidine | 986 | 5.0 | 0.930 | 4.650 | 350 | 0.7 | 14 | 1.4 | 182 | 0.0 |
| Sulfadimidine | 1283 | 5.0 | 0.930 | 4.650 | 350 | 0.7 | 14 | 1.4 | 182 | 0.0 |
| Sulfadimidine | 1284 | 5.0 | 0.930 | 4.650 | 350 | 0.7 | 14 | 1.4 | 182 | 0.0 |
| Sulfadimidine | 1243 | 30.0 | 0.930 | 27.900 | 300 | 0.8 | 4 | 29.1 | 182 | 0.2 |
| Sulfadimidine | 1244 | 30.0 | 0.930 | 27.900 | 300 | 0.6 | 4 | 38.8 | 182 | 0.2 |
| Sulfadimidine | 1122 | 100.0 | 0.930 | 93.000 | 600 | 1.5 | 5 | 20.7 | 182 | 0.1 |
| Sulfadimidine | 1256 | 10.0 | 0.930 | 9.300 | 240 | 0.2 | 5 | 38.8 | 182 | 0.2 |
| Sulfadimidine | 1116 | 100.0 | 0.930 | 93.000 | 400 | 0.9 | 5 | 51.7 | 182 | 0.3 |
| Sulfadimidine | 1118 | 100.0 | 0.930 | 93.000 | 400 | 1 | 5 | 46.5 | 182 | 0.3 |
| Sulfadimidine | 1197 | 5.0 | 0.930 | 4.650 | 100 | 0.9 | 5 | 10.3 | 182 | 0.1 |
| Sulfadimidine | 1183 | 60.0 | 0.930 | 55.800 | 1000 | 0.9 | 5 | 12.4 | 182 | 0.1 |
| Sulfadimidine | 1185 | 15.0 | 0.930 | 13.950 | 1000 | 0.2 | 5 | 14.0 | 182 | 0.1 |
| Sulfaquinoxaline | 1289 | 120.0 | 0.150 | 18.000 | 2000 | 0.8 | 5 | 2.3 | 60 | 0.0 |
| Trimethoprim | 1264 | 100.0 | 0.020 | 2.000 | 650 | 0.7 | 5 | 0.9 | 6 | 0.1 |
| Trimethoprim | 1293 | 60.0 | 0.080 | 4.800 | 150 | 0.7 | 7 | 6.5 | 6 | 1.0 |
| Trimethoprim | 1294 | 60.0 | 0.080 | 4.800 | 200 | 0.9 | 10 | 2.7 | 6 | 0.4 |
| Trimethoprim | 1295 | 60.0 | 0.080 | 4.800 | 150 | 1.1 | 7 | 4.2 | 6 | 0.6 |
| Trimethoprim | 1091 | 45.0 | 0.050 | 2.250 | 300 | 0.4 | 5 | 3.8 | 6 | 0.6 |
| Trimethoprim | 1111 | 45.0 | 0.050 | 2.250 | 300 | 0.5 | 5 | 3.0 | 6 | 0.5 |
| Trimethoprim | 1113 | 15.0 | 0.050 | 0.750 | 100 | 0.5 | 5 | 3.0 | 6 | 0.5 |
| Trimethoprim | 1181 | 150.0 | 0.050 | 7.500 | 500 | 1.4 | 5 | 2.1 | 6 | 0.3 |
| Tylosin | 1190 | 15.0 | 0.045 | 0.675 | 250 | 0.08 | 3 | 11.3 | 81 | 0.1 |
| Tylosin | 1191 | 15.0 | 0.045 | 0.675 | 250 | 0.08 | 3 | 11.3 | 81 | 0.1 |
| Tylosin | 1192 | 15.0 | 0.045 | 0.675 | 250 | 0.08 | 3 | 11.3 | 81 | 0.1 |
| Tylosin | 1189 | 120.0 | 0.045 | 5.400 | 200 | 1.4 | 2 | 9.6 | 81 | 0.1 |
| Tylosin | 1092 | 45.0 | 0.182 | 8.190 | 300 | 0.4 | 5 | 13.7 | 81 | 0.2 |
| Tylosin | 1110 | 45.0 | 0.182 | 8.190 | 300 | 0.5 | 5 | 10.9 | 81 | 0.1 |
| Tylosin | 1112 | 15.0 | 0.182 | 2.730 | 100 | 0.5 | 5 | 10.9 | 81 | 0.1 |
| Tylosin | 999 | 5.0 | 0.090 | 0.450 | 146 | 0.6 | 5 | 1.0 | 81 | 0.0 |
| Tylosin | 1260 | 30.0 | 0.090 | 2.700 | 150 | 0.7 | 5 | 5.1 | 81 | 0.1 |
| Tylosin | 1263 | 30.0 | 0.090 | 2.700 | 200 | 0.4 | 5 | 6.8 | 81 | 0.1 |
| Tylosin | 1211 | 400.0 | 0.090 | 36.000 | 5000 | 1.4 | 3 | 1.7 | 81 | 0.0 |

Abbreviations: AS = active substance; UDD = used daily dose; DDDvet = defined daily dose; ESCAC = European Surveillance of Veterinary Antimicrobial Consumption

Supplementary Table 19: Evaluation of dosage for all treatments

|  | **N** | **%** |
| --- | --- | --- |
| **dosage** |  |  |
| **under** | 144 | 87.80 |
| **normal** | 8 | 4.88 |
| **over** | 12 | 7.32 |
| **All** | 164 | 100.00 |

Abbreviations: N = # of treatments

Supplementary Table 20: Evaluation of dosage by ingredient

|  | **active substance** | | | | | | | | | | | | | | | | | | | | | | | | |
| --- | --- | --- | --- | --- | --- | --- | --- | --- | --- | --- | --- | --- | --- | --- | --- | --- | --- | --- | --- | --- | --- | --- | --- | --- | --- |
|  | **Amoxicillin** | | **Doxycycline** | | **Enrofloxacin** | | **Flumequin** | | **Neomycin** | | **Oxytetracycline** | | **Sulfachlorpyridazine** | | **Sulfadiazine** | | **Sulfadimidine** | | **Sulfaquinoxaline** | | **Trimethoprim** | | **Tylosin** | | **All** |
|  | **N** | **%** | **N** | **%** | **N** | **%** | **N** | **%** | **N** | **%** | **N** | **%** | **N** | **%** | **N** | **%** | **N** | **%** | **N** | **%** | **N** | **%** | **N** | **%** | **N** |
| **dosage** |  |  |  |  |  |  |  |  |  |  |  |  |  |  |  |  |  |  |  |  |  |  |  |  |  |
| **under** | . | . | 17 | 77.3 | 10 | 66.7 | 1 | 100.0 | 3 | 100.0 | 68 | 94.4 | 3 | 75.0 | 4 | 100.0 | 19 | 100.0 | 1 | 100.0 | 7 | 87.5 | 11 | 100.0 | 144 |
| **normal** | 1 | 25.0 | 1 | 4.5 | 4 | 26.7 | . | . | . | . | . | . | 1 | 25.0 | . | . | . | . | . | . | 1 | 12.5 | . | . | 8 |
| **over** | 3 | 75.0 | 4 | 18.2 | 1 | 6.7 | . | . | . | . | 4 | 5.6 | . | . | . | . | . | . | . | . | . | . | . | . | 12 |
| **All** | 4 | 100.0 | 22 | 100.0 | 15 | 100.0 | 1 | 100.0 | 3 | 100.0 | 72 | 100.0 | 4 | 100.0 | 4 | 100.0 | 19 | 100.0 | 1 | 100.0 | 8 | 100.0 | 11 | 100.0 | 164 |

Abbreviations: N = # of treatments

## Supplementary Figures


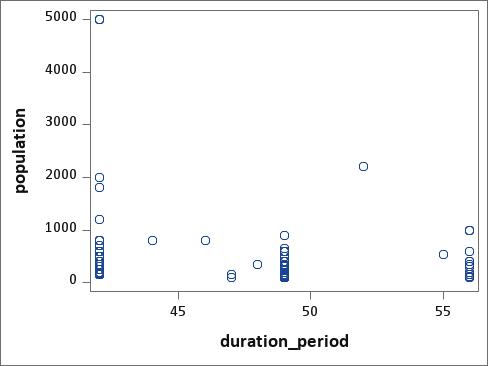

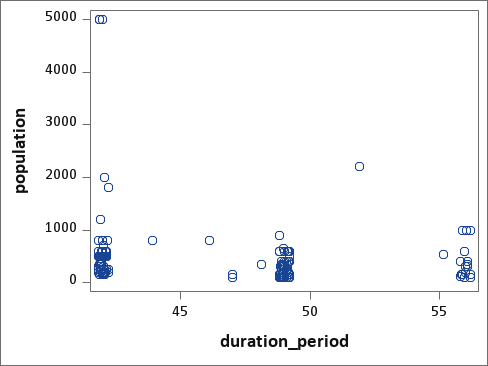


A B

**Supplementary Figure 1.** Scatter-plots without jittering (A) and with jittering (B) : jittering to prevent overplotting/specifies that data markers are offset when multiple observations have the same response value
